# Decreased attention in 10- and 14-month-olds with neurofibromatosis type 1 and association with later ADHD traits

**DOI:** 10.1186/s11689-026-09702-3

**Published:** 2026-05-09

**Authors:** Tessel Bazelmans, Francesca Penza, Jannath Begum-Ali, Chloe Taylor, Mark H. Johnson, Tony Charman, Jonathan Green, Shruti Garg, Emily J. H. Jones, STAARS and EDEN Teams, Mary Agyapong, Leila Dafner, Mutluhan Ersoy, Laurel Fish, Teodora Gliga, Amy Goodwin, Rianne Haartsen, Hanna Halkola, Alexandra Hendry, Rebecca Holman, Sarah Kalwarowsky, Anna Kolesnik, Sarah Lloyd‐Fox, Luke Mason, Nisha Narvekar, Greg Pasco, Laura Pirazzoli, Grace Vassallo, Emma Burkitt‐Wright, Judith Eelloo, D Gareth Evans, Siobhan West, Eileen Hupton, Lauren Lewis, Louise Robinson, Angus Dobbie, Ruth Drimer, Saghira Malik Sharif, Helen Bethell, Rachel Jones, Susan Musson, Catherine Prem, Miranda Splitt, Karen Horridge, Diana Baralle, Carolyn Redman, Helen Tomkins

**Affiliations:** 1 Centre for Brain and Cognitive Development, Birkbeck, University of London, London, UK; 2 Department of Psychology, University of Cambridge, Cambridge, UK; 3 Department of Psychology, Institute of Psychiatry, Psychology & Neuroscience, King’s College London, London, UK; 4 Division of Psychology & Mental Health, School of Health Sciences, University of Manchester, Manchester, UK; 5 Royal Manchester Children’s Hospital, Manchester University NHS Trust, Manchester, UK; 6 MRC Centre for Developmental Neurobiology and Department of Child and Adolescent Psychiatry, Institute of Psychiatry, Psychology & Neuroscience, King’s College London, London, UK

**Keywords:** Focused attention, Vigilance, Sustained attention, Movement, ADHD, Neurofibromatosis, Neurodevelopmental conditions, Infants

## Abstract

**Background:**

Identifying precursors to ADHD, which affects up to 5% of children, is crucial for early identification and support. To this end, we used a prospective sample to investigate endogenous attention and activity level in infants with and without an elevated likelihood (EL) for ADHD and investigated associations with ADHD traits at 3-years. EL status was based on a family history of autism and/or ADHD or a diagnosis of neurofibromatosis type 1 (NF1), a genetic condition associated with higher rates of ADHD.

**Methods:**

Infants (*n =* 26 typical likelihood (TL), *n =* 70 EL-autism, *n =* 28 EL-ADHD, *n =* 18 EL-autism + ADHD, and *n =* 29 NF1) participated in a live puppet task at 10 and/or 14 months. Mixed-effect models compared groups on behaviourally coded Focused Attention and Vigilance (i.e. Sustained Attention) and Movement, which was measured concurrently using an accelerometer to capture activity during these distinct attention states. Finally, we examined the bivariate associations of Attention and Movement, and their interaction, with 3-year parent-reported ADHD traits.

**Results:**

Infants with NF1 exhibited less Focused Attention and Vigilance than EL-ADHD [t (1,170) = 3.53, p = .005] or EL-autism [t (1,170) = 5.43, *p* < .001] infants and this did not differ across age. There were no Attention differences between the EL and TL groups and no Group or age differences in Movement, however Movement did vary by Attention type: ${\chi }^{2}$(2) = 215.25, *p* < .001 (Focused Attention < Vigilance < Looking elsewhere). Across the cohort, less Focused Attention at 10 months (rs = -.27, p = .008) was associated with more ADHD traits at 3-years. During Vigilance at 14 months, there was a significant Attention-by-Movement interaction effect (*z* = 25.65, *p* < .001), showing that the association between more Vigilance and fewer ADHD traits was most pronounced in infants showing less Movement.

**Conclusions:**

Reduced attention was observed from 10 months onwards in those with NF1, but not in those with a familial likelihood of ADHD. Moreover, early focused attention and the ability to modulate activity level by attentive state (i.e. more vigilant, less movement) may be important emerging features associated with later ADHD traits. We consider implications for early detection and early support strategies.

**Supplementary Information:**

The online version contains supplementary material available at 10.1186/s11689-026-09702-3.

## Introduction

Attention Deficit Hyperactivity Disorder (ADHD) is a neurodevelopmental condition characterised by inattention, hyperactivity and impulsivity that can affect school attainment, mental health and quality of life [1]. ADHD affects approximately 3—5% of the population [2, 3]. According to the diagnostic criteria, behavioural symptoms of ADHD emerge before the age of 12 years [1]. For most children, symptoms are present by age 7 years [4], although overall there is continuity in symptoms from 3—4 years to mid-childhood [5–7]. Due to the long-term negative effects on individuals’ quality of life [8], early ADHD diagnosis and treatment are imperative yet seldom occur in practice [9]. Therefore, identifying precursors and understanding the causal pathways leading to ADHD can further inform the development and provision of early interventions [10].

ADHD is a substantially heritable condition [11] and having a diagnosed sibling or parent increases the likelihood of having a diagnosis by around 5 to 13 times [12, 13]. Moreover, ADHD often co-occurs with other neurodevelopmental conditions such as autism within the same child [13], or within the family [12]. Therefore, to investigate the causal pathways leading to neurodevelopmental conditions such as ADHD, researchers have adopted the ‘infant sibling’ design, which allows for the longitudinal follow-up of infants with a family member with an ADHD and/or autism diagnosis. This approach has often been used in prospective studies of autism [14, 15], but only more recently in ADHD (e.g. [16–18]). However, a well-recognised limitation of these designs is that they may not fully capture the onset patterns of ADHD associated with more penetrant monogenic conditions.

This limitation can be ameliorated by using complementary prospective studies of infants with genetic conditions linked to an increased likelihood of ADHD, such as neurofibromatosis type 1 (NF1) [11, 19]. NF1 is a monogenic condition that affects around 1 in 2000 to 3000 infants [20, 21] and can be identified early in development through clinical features or cord blood testing for the familial pathogenic variant. In addition to its physical phenotype, many children with NF1 experience behavioural, cognitive, emotional and psychosocial difficulties [22], with around half meeting diagnostic criteria for ADHD [23]. Recently, prospective studies of familial likelihood of ADHD have been complemented with longitudinal NF1 studies [24]. This combined monogenic and polygenic approach expands the understanding of developmental pathways by offering the opportunity to identify early neurocognitive and behavioural markers of ADHD traits in a more diverse population than only those with a familial likelihood. Further, it provides the opportunity to determine which early differences are shared across or specific to mono and polygenic forms of ADHD.

Most research on early precursors of ADHD has focused on attention and activity level, as these are the primary two dimensions of the diagnostic criteria. In infancy, attention and activity levels are often measured using parent reports, behavioural observations during free play and experimental methods (e.g. eye-tracking, accelerometer, motion tracking) [18, 25, 26]. Using such methods, some prospective studies of premature infants have shown associations between poorer early focused attention and later ADHD traits [27, 28]. Focused attention is commonly defined as the ability to maintain attention on a specific stimulus or event [29]. For example, Lawson & Ruff (2004) showed that lower levels of focused attention on a toy at 7 months (looking whilst manipulating) predicted more ADHD traits at 4—5 years [27]; and Ruff et al. showed that reduced focused attention at 12 months predicted more ADHD traits at 3.5 years in preterm but not full-term infants [28].

A range of other prospective studies have identified clearer differences in activity level than in attention. Auerbach et al. reported that infants with a family history of ADHD had higher parent-rated activity level at 7, 12 and 25 months than infants with no family history of ADHD [30]. However, attention differences were only observed at 7 months; they did not examine the relation to later ADHD traits. Further, Miller et al. report that infants with a family history of ADHD showed increased impulsivity/activity level during a behavioural assessment on examiner-rated behaviour from 12 months, but did not show differences on moment by moment coding of attention at 12, 18 or 24 months [18]. In a follow up, Miller et al. showed that a latent class of familial likelihood children with high ADHD traits at 3 years showed differences in inattention during a behavioural assessment at 24 months but not 12 months [31]. Similarly, in the same familial likelihood sample as presented here, we did not find early differences in attention (measured using parent report, patterns of looking to toys and eye-tracking tasks) in infants with an elevated likelihood for ADHD and no associations with 3-year ADHD traits [25]. There was, however, a relationship between parent and experimenter reported activity level at 10 months and later ADHD traits [25]. Using a more objective measure of movement such as an accelerometer, Reetzke et al., reported that infants with a familial history of ADHD and autism showed higher activity levels at 18 months, but not at 12 months [26]. Taken together, these findings suggest that group differences in activity level are relatively consistently detected from infancy, especially in family history samples, but early differences in attention are less clear.

Not many studies have yet focused on early attention differences in NF1 infants; however, in the same longitudinal cohort of infants as in the present study, our research group reported differences in visual attention using eye-tracking tasks. Specifically, NF1 infants showed slower development of saccadic reaction times and of visual foraging from 10 to 14 months compared to both infants with and without family history of ADHD [24]. However, these tasks did not measure focused or vigilant attention.

One challenge in the interpretation of this literature is that different facets of attention and activity level are often measured using different tasks, through different approaches or in different populations. Thus, it is difficult to determine whether some tasks are more sensitive than others, whether different aspects of attention are more sensitive to emerging ADHD, whether early signs of ADHD differ by etiological group (e.g. premature infants, monogenic conditions or family history) or whether there are true differences in the developmental timing of the emergence of differences in attention and activity level. Further, sometimes the definition of attention encompasses information about movement (e.g. an infant might move away from a toy, thus increasing their level of motion and decreasing their level of attention). Thus, to test the emergence of differences in activity level and attention in the emergence of ADHD traits it is necessary to use approaches that can measure activity level and attention within the same task, but where the two domains can be separated.

Further, within the umbrella term of attention, an important distinction can be made between focused attention to a particular stimulus, and maintaining attention during periods of less input, sometimes called sustained attention or vigilance [29]. Often, children with ADHD have greatest difficulty with this latter kind of attention, making it important to study [32–34]. One paradigm that separates both facets of attention is a puppet task developed by Ruff et al. [35]. In this task, a puppet appears and disappears behind a curtain. Focused attention is defined as infants’ looking behaviours to the puppet when it was visible and vigilance as their looking behaviours to the place where a puppet had previously appeared, i.e., during the inter-stimulus interval. They found that 5-month-old infants showed evidence of maintaining attention to the place where they expected the puppet to appear (vigilance), which increased at 9 and 11 months [35].

Here, we used the puppet task to examine Focused Attention, Vigilance as well as Movement in a sample with an elevated likelihood (EL) of ADHD traits by virtue of having a familial history of ADHD and/or autism or having an NF1 diagnosis. We focused on 10 and 14 month as individual differences in attention starting from the second half of the first year have been related to later ADHD-related outcomes [36]. More specifically, we assessed Focused Attention (i.e., attention to the puppet stimulus) and Vigilance (i.e., attending to where the puppet previously appeared) using behavioural coding; Movement was concurrently measured using an accelerometer to provide an objective measure of activity levels during the two types of attention and when the infant was not attending.

The first objective was to assess group differences in Focused Attention, Vigilance and Movement between infants with an elevated likelihood (EL) for ADHD (i.e., a familial likelihood of ADHD and/or autism or a diagnosis of NF1) and infants at typical likelihood (TL). We hypothesised that infants in the EL for ADHD groups would show less Focused Attention, less Vigilance and more Movement during periods of attention compared to TL. The second objective was to test whether Attention and Movement at 10 and/or 14 months were associated with ADHD traits at 3 years of age across the full sample. We hypothesised that less Focused Attention and Vigilance and more Movement would be associated with greater parent-reported ADHD traits at 3 years. Lastly, we hypothesised that the combination of lower Attention and greater Movement in infancy would be associated with the highest ADHD traits at age 3 years.

## Methods

### Participants

Infants with a family history of neurodevelopmental conditions were part of an ongoing longitudinal study STAARS (Studying Autism and ADHD in the eaRly yearS), which started in 2013. Here, we include infants who were recruited up till 2019 (*n =* 187). Infants were enrolled in the study if they had a first-degree relative with a diagnosis or probable diagnosis of ADHD (EL-ADHD, *n =* 31), a first-degree relative with a diagnosis of autism (EL-autism, *n =* 78) or if both diagnoses were present in one or more family members (EL-autism + ADHD, *n =* 20). Infants who had no first-degree relatives with either autism or ADHD were enrolled as typical likelihood (TL, *n =* 27) children. The NF1 cohort (*n =* 31) was part of the Early Development in Neurofibromatosis Type 1 (EDEN) research project, and we included infants with a research visit prior to January 2020.

Before enrolment, families were interviewed using a telephone screening form to assess the family history status, which was confirmed at their first in-person visit. Our protocol used for classification of familial status of autism and ADHD was previously reported [16, 25]. The presence of ADHD was defined as infants with a first-degree relative (parent or sibling) with a community clinical diagnosis of ADHD. Parent’s historical diagnosis was sufficient for inclusion. If parents reported concerns about ADHD traits in the family and the parent or older sibling did not have a community clinical diagnosis of ADHD, screening questions were used to determine the probable presence of ADHD (see Supplemental Materials (SM)1 for details on parent/sibling diagnosis vs screening). Screening was done using a shortened version of the Conners Early Childhood [37] (siblings < 6 years), Conners 3 (siblings > 6 years), or Conners Adults ADHD Rating Scale (CAARS). This shortened version included the items that are part of the symptom count for DSM ADHD symptoms, on which we based our inclusion criteria. Thresholds for inclusion were the presence of four (Conners Early Childhood) or five (Conners 3) [38] ADHD traits on either the Hyperactivity/Impulsivity or Inattention scale and a positive score on the impairment scale. For parents, the threshold for inclusion was the presence of five ADHD traits on either the Hyperactivity/Impulsivity or Inattention scale. This screening protocol is consistent with those used in other studies of elevated ADHD likelihood [39].

In the EL-autism group, all infants had at least one older sibling with a clinical community diagnosis of autism. The EL-autism + ADHD group met criteria as described above for both the EL-autism and EL-ADHD groups.

The TL infants all had at least 1 older sibling and no first-degree relative with a diagnosis of autism or ADHD at enrolment. This was asked during screening and confirmed at their first visit. They were recruited from a volunteer database at the Centre for Brain and Cognitive Development, Birkbeck, University of London. Inclusion criteria included full-term birth (gestational age > 36 weeks) and none of the infants had a known medical or developmental condition at the time of enrolment.

Infants with NF1 were recruited via local and regional genetic centres (Manchester, Leeds, Newcastle, and Southampton) and via advertisements placed in the NF charities’ social media webpages. The EDEN study has Research & Development approval for recruitment across all specialists’ genetic centres across the UK, and information about the study was offered to eligible participants at routine clinical appointments. Within the general population, NF1 is approximately 50% familial and 50% de novo [40]. Our sample consists primarily of familial cases as these are typically identified earlier in development through routine cord blood testing within the state-funded health service. Of note, our previous behavioural phenotyping studies have shown no differences between familial and de novo cases [41]. All the participants who had inherited NF1 were confirmed via molecular testing of cord blood samples or clinical diagnosis based on NIH consensus criteria [42].

**Fig. 1:**
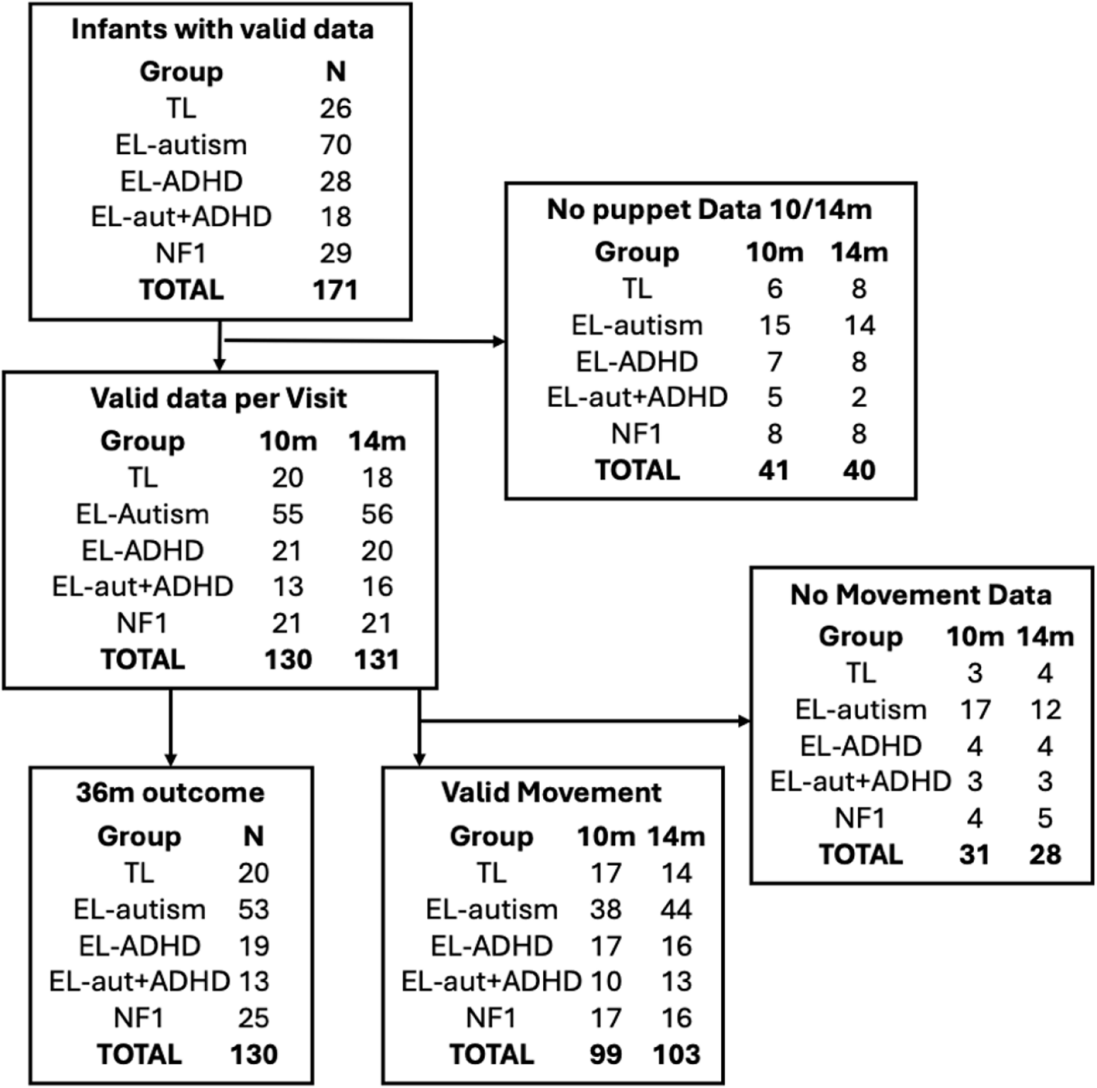
Flowchart of included and excluded participants for different analyses. EL-aut + ADHD = EL-autism + ADHD group

The final dataset consisted of 171 infants (*n =* 26 TL, *n =* 70 EL-autism, *n =* 28 EL-ADHD, *n =* 18 EL-autism + ADHD, *n =* 29 NF1, see Fig. 1 for flowchart) with valid behavioural data from the puppet live task at 10 (*n =* 130) and/or 14 months (*n =* 131). Of the 171 infants, 90 (53%) had valid behavioural data at both visits (see SM2 for availability of data by Timepoint and measure and SM3 for comparison of participants with and without available data).

Ethical approval was granted by the National Research Ethics Service and the Research Ethics Committee of the Department of Psychological Sciences, Birkbeck, University of London. Informed written consent was provided by the parent(s) prior to the commencement of the study. The in-person testing sessions for both the STAARS and EDEN studies was conducted by the same testing team and only took place if the infants were content and alert. Participant families were reimbursed for travel, subsistence, and overnight stay if required.

### Stimuli

**Fig. 2:**
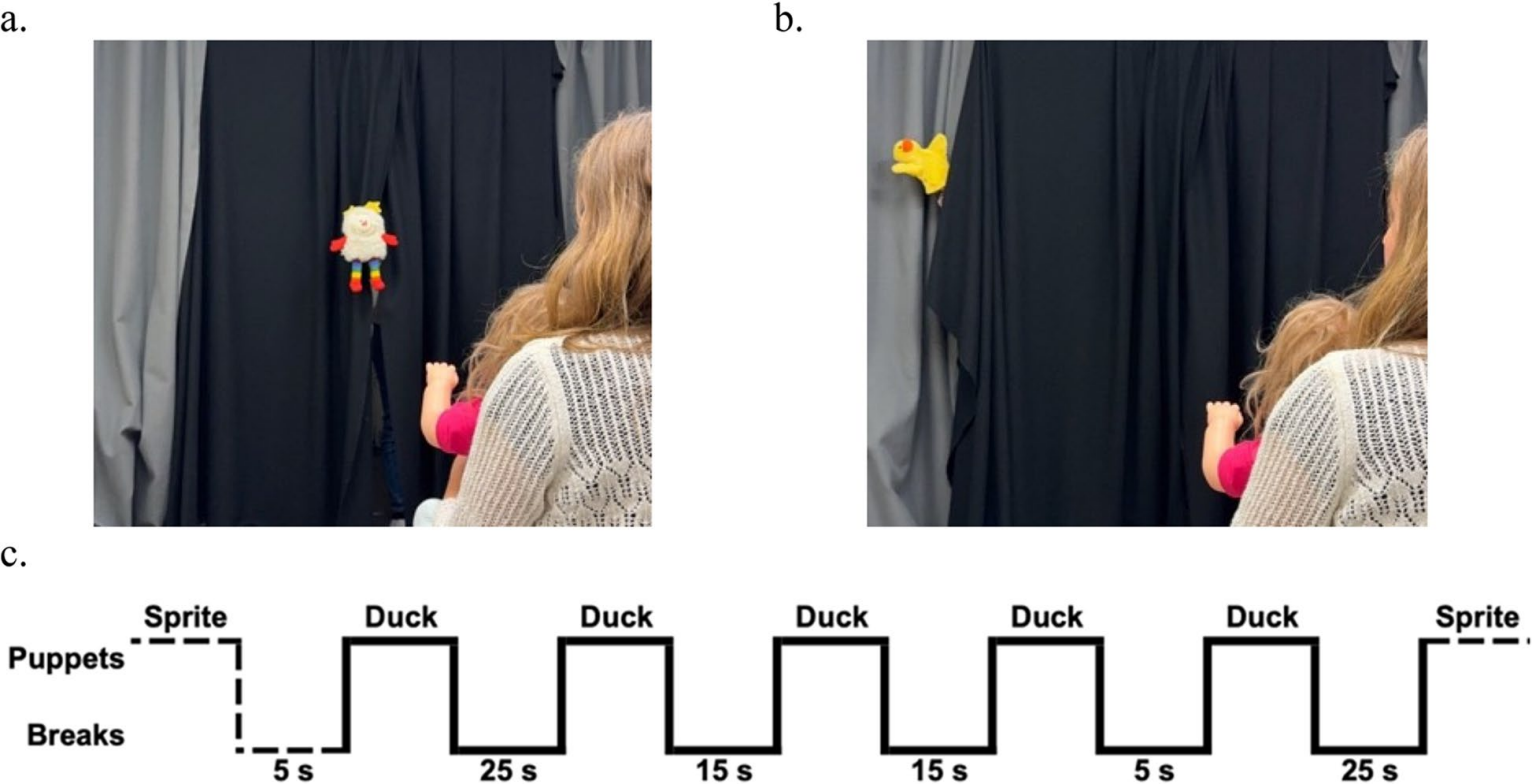
**a** Sprite; **b** Duck; **c** Timeline. Puppets: 4—8 s. Dashed lines are not included in the analyses

The task was adapted from the paradigm originally described by Ruff et al. [35]. Infants (10 months: *n =* 101 and 14 months: *n =* 93) were seated in a highchair (55 cm in height) or on their parent’s lap (chair 39–55 cm in height, 10 months: *n =* 29 and 14 months: *n =* 38) approximately 2.1–2.5 m in front of a black screen (127.5 × 189.5 cm) made up of two pieces of black cloth (see Fig. 2a and b). A white sprite doll (27 × 31 cm) held by the experimenter was then shown in the upper-middle of the screen (at a height of approx. 137 cm) while the experimenter said “Hi < infant’s name >, I’m a sprite” and moved the doll left and right around its sagittal axis for 4–8 s. The experimenter then withdrew the sprite back behind the screen. After a five-second no puppet interval (break), the experimenter then held a yellow duck puppet (23 × 20 cm) to the left (as seen by the infant) of the screen (at a height of approx. 150 cm) and said “Hi < infant’s name >, hello, hi” while making the puppet wave its hands and nod its head for 4–8 s (‘puppets’). The experimenter then withdrew the puppet behind the screen. The duck trial was repeated four more times with breaks of 25, 15, 15, and 5 s (‘breaks’), matching the times of the original task [35]. After the fifth duck trial, there was a 25-s break before the sprite trial was repeated for a second time (see Fig. 2). The data included in this manuscript start at the first duck trial and end after the last (Fig. 2c; see SM4 for average durations of each puppet and breaks across infants). Three custom-built cameras simultaneously recorded the puppet show and the infant’s looking behaviour at a rate of 25 fps. The parent was allowed to comfort the infant if they became upset. Infants were excluded from the analysis if the parent moved them from the highchair to their lap during the puppet show, if the infant was too upset to continue with the show, or if there was a technical issue (e.g., cameras not working or incorrect number of puppets shown). Only those infants with valid data for the full puppet paradigm were included.

### Attention coding scheme

The infant’s looking behaviour was coded in Mangold INTERACT v16 and was divided into three categories: looking at the puppet (Focused Attention); looking at where the puppet was (during the no puppet intervals; Vigilance); and looking elsewhere. Throughout the puppet show, one camera recorded the black screen, and the other two recorded the infant’s face. The separate videos were played back simultaneously in the coding software, allowing the coder to establish the angle of looking for both the sprite doll and the duck puppet for each infant. The infant’s looks towards where the puppet was after each puppet interval could then be coded based on these angles. Each look was recorded inclusively from the first frame to the last frame of when the infant was looking in the specified direction. If the infant blinked and continued to look in the same direction this was counted as a continuous look. All the videos were coded at a playback speed of 17.5 fps by one researcher (CT) and 10% were randomly selected and double-coded by a second researcher who was blind to the group status of each infant. Intraclass correlations (two-way mixed; single measures; absolute agreement) were calculated for each puppet and each break separately and were 0.908 and above (see SM5 for details). We extracted two key variables from the behavioural coding files: 1. *Focused Attention:* The amount of time the infants spent looking at each puppet while it was present, calculated as a proportion of the puppet’s presentation duration and averaged across all puppet trials (ranged from 0.34 to 1; see also SM6); 2. *Vigilance* (i.e., the ability to remain alert to unexpected or rare events): The amount of time the infants spent looking at where the puppet previously appeared during each inter-puppet interval, calculated as a proportion of the duration of the interval when no puppet was visible for that presentation and then averaged across breaks (ranged from 0.01 to 0.72). All included infants had data available for the full paradigm of five puppets and five breaks.

### Movement

Movement data was collected during the puppet task along three axes in *g*, gravity force, units using an accelerometer (BN-ACCL3) attached to the child’s upper left thigh, wirelessly connected to the Biopac MP150 system and recorded using AcqKnowledge 4.4 at either 100 or 1000 Hz (BIOPAC Systems, Inc, Goleta, CA, USA). All data was down sampled offline to 100 Hz. An LED light was used to sync the accelerometer and video data. This LED light was coded in Mangold as part of the coding scheme and appeared as a digital marker in the AcqKnowledge file when turned on and off. Accelerometer data was imported into Matlab R2019a and the correct segment for the Puppet task was selected based on the LED markers. A 5 Hz 3rd order low-pass Butterworth filter was applied to each individual axis. Next, axes were combined using Pythagoras (x^2^ + y^2^ + z^2^). A 1 Hz 3rd order high-pass Butterworth filter was applied to remove the constant effect of gravity from the combined accelerometery data. Any values between −0.01 and 0.01 were treated as no Movement and set to 0. Using the timestamps from Mangold, average Movement data was extracted for each period and type of behaviour. We divided Movement behaviour based on type of attention: 1. *Movement Focused Attention:* the average amount of Movement when looking at a puppet during puppets; 2. *Movement Vigilance:* the average amount of Movement when looking at where the puppet was during breaks. 3; *Movement Looking Elsewhere*: the average amount of Movement when not in Focused Attention or Vigilance). The mean of the absolute accelerometer values was taken and multiplied by 981 to yield movement in cm/s^2^. In total, 41 children (10 m: *n =* 25; 14 m: *n =* 16) did not have accelerometer data available, which was primarily due to issues with equipment (technical difficulties with Biopac/accelerometer) or issues with syncing the video and accelerometer data (e.g. LED flash not recorded on video). Of the 153 infants with Movement data, 49 (32%) had data at both visits (see SM2 for details per data type and visit).

### Cognitive and adaptive functioning and motor skills

The Mullen Scales of Early Learning (MSEL) [43] is a developmental assessment of cognitive functioning in infants and preschool-aged children. The Visual Reception, Fine Motor, Receptive Language and Expressive Language scales together make up the Early Learning Composite (ELC). All subscales, including the Gross Motor scale, of the MSEL were administered at 10 and 14 months by trained researchers from the STAARS team (see SM7 for administration details). The Vineland Adaptive Behavior Scales – second edition (VABS) [44] parent questionnaire version was used to measure adaptive functioning across Socialization, Communication, Daily Living Skills, and Motor Skills domains. Items in each domain are scored from 0 (never) to 2 (usually). The domains of this standardized assessment tool can be combined into the Adaptive Behaviour Composite (ABC). The MSEL-ELC and VABS-ABC each have a mean of 100 and SD of 15. A combined Motor Skills score was created based on the z-scored fine and Gross Motor scales of the MSEL and the Motor Skills domain of the VABS. Combining the scales was motivated by the significant correlations between the three domains (all *r*’s > 0.19, all *p*’s £ 0.041) and individual scales were highly correlated with the combined Motor Skills score (all *r*’s > 0.64, all p’s £ 0.001; see SM8).

### ADHD traits

The Child Behaviour Checklist (CBCL-1.5—5 years, [45]) DSM-oriented ADHD scale, consisting of 6 items (score range 0 to 12), was used to measure ADHD traits at age 3 years. Parents reported their children’s behaviours within the last two months on a 3-point Likert scale (0 “Not True” to 1 “Somewhat or Sometimes True” to 2 “Very True or Often True”). The total score ranges from 0 to 12, with a score of 10 or higher considered to be in the (borderline) clinical range. At 3 years, 130 (76%) infants had ADHD questionnaire data available (also see SM2, Table S2.1).

### Analysis

Descriptive analyses, linear mixed models and associations with 3-year traits were conducted in Stata 19.0 [46]. Mixed model Beta-regression was performed in SAS Studio (https://welcome.oda.sas.com). Group differences in sex, age in months, and developmental abilities (Motor Skills, ELC, and ABC) were assessed using Chi-square or ANOVA, followed by Tukey–Kramer post-hoc comparisons.

Inspection of variable distributions showed that Focused Attention displayed a negatively skewed distribution, whereas Vigilance and Movement data showed a positive skew. Looking data was transformed using a logit transformation; Movement data was transformed using a natural log transformation. Before transformation, 0 and 1 values in Looking and Movement data were adjusted by adding or subtracting 0.01. Transformation improved the normal distribution of the data in general, however the cases previously having 0 or 1 values were still observed as outliers. We report in text where untransformed versus transformed variables were used.

As infants during the puppet task were sitting either on their parents’ lap or a highchair, we used ANOVAs to assess whether there were differences in Attention and Movement due to Position as well as the Position-by-Group and Position-by-Condition interaction effects. Considering the potential effect of Motor Skills on Movement and of Position on Attention and Movement, if significant Group differences were found, analyses were repeated including Motor Skills and/or Position as control variables.

#### Attention

First, we used Spearman’s correlations to assess the association between Focused Attention and Vigilance. Next, we compared Attention behaviour of the five groups including Condition (Focused Attention and Vigilance) and the two Timepoints (10 and 14 months) as repeated measures. We ran a mixed-effects beta regression, a subtype of generalized linear mixed models (GLMMs), to look at the effect of Group (TL, EL-ADHD, EL-autism, EL-autism + ADHD and NF1), Condition (Puppet vs Breaks) and Timepoint (10 vs 14 months) and their interaction effects. This statistical approach handles non-normally distributed data such as continuous proportion data that are bounded between 0 and 1 and which often cluster toward one end of the range. The mixed-effects beta regression model was run using the *proc glimmix* command in SAS. We used a logit function for the Attention data, included Subject as a random intercept and fitted an unstructured covariance accounting for within-subject variability; the model including the random intercept of Timepoint, to consider that observations of each Subject are also nested within Timepoint, did not converge. Odds Ratios (OR) were calculated as a measure of effect size. To look at the robustness of the finding, we repeated the analysis for the Attention data by conducting a linear mixed model in STATA (mixed command; see SM9 for details) on the transformed data, which led to comparable results.

#### Movement

Next, to compare the five Groups on their Movement during the three Conditions (Focused Attention, Vigilance and Looking Elsewhere), we first fitted an inverse Gaussian GLMM in SAS but the model did not converge. We subsequently ran a linear mixed model in STATA on the transformed Movement data (See SM6), including Group (TL, EL-ADHD, EL-autism, EL-autism + ADHD and NF1), the repeated measures Condition (Focused Attention, Vigilance and Looking elsewhere) and Timepoint (10 vs 14 months) and their two-way and three-way interactions as predictor variables. Subject and Timepoint (nested within Subject) were added as random intercepts to account for intra-individual correlation across all conditions and for session-specific variability within each Timepoint.

We used maximum likelihood and robust standard errors due to the heteroscedasticity of the residuals and independent residual structure. Scatterplots of fitted values and residuals, as well as Q-Q plots of residuals and best linear unbiased predictors (BLUP) for random effects of ID, were inspected to evaluate the model fit [47]. Analyses were repeated excluding participants with standardised residuals |> 3|. The effect of categorical predictor variables was assessed using the contrast command and significant effects were compared post-hoc using margins, pwcompare and corrected using Bonferroni. Any significant interaction was followed up by calculating estimated marginal means (EMMs) per Condition to interpret the effect. Effect sizes for significant predictors were determined by calculating the change in total variance explained by fixed effects (marginal R^2^) using the r2_mlm command [48] and by exponentiating model coefficients to estimate the proportional changes in movement. Due to transformation issues and to confirm the robustness of our findings, we repeated the analysis on the untransformed data using a non-parametric Kruskal–Wallis test for Condition and Group separately, followed by pairwise Mann–Whitney comparisons.

#### Longitudinal associations with ADHD traits at 3 years

Next, to examine the association with 3-year ADHD traits, we first ran bivariate Spearman’s correlations with the untransformed Focused Attention, Vigilance and Movement data at 10 and 14 months, separately. Lastly, we looked at the interaction between Attention and Movement to examine if a combination of decreased Attention and increased Movements was associated with the highest levels of ADHD traits at age 3 years. We modelled each Timepoint and Condition (Focused Attention, Vigilance) separately, resulting in four Negative Binomial regression models to fit the count data of the CBCL and avoid overdispersion. Each model included the Attention-by-Movement interaction effect which was the main variable of interest. Attention data was centered and Movement data was transformed and centered to aid the interpretation of any significant interaction. To estimate the effect size, we calculated incident rate ratios (IRRs) for the interaction term and probed significant interaction by estimating conditional simple slopes at -/+ 1 SD of movement. Influential points were identified using Cook’s Distance (threshold = 4/*n*) and significant models were re-rerun excluding influential points.

## Results

### Included versus excluded infants

Comparison of included (valid puppet data) and excluded (no/invalid puppet data but attended the visit) infants showed that participants included at 14 months had lower Vineland ABC score compared to the excluded children (94 vs 99, *t* = 2.23, *p* = 0.028). There were no age, sex, motor or other cognitive differences at either 10 or 14 months (all *p* > 0.130). Infants with or without Movement data did not differ on measures at 10 or 14 months (all *p* > 0.087; see SM3 for details).

### Final sample descriptives

Table 1 provides information about participants’ demographic and developmental abilities collected at 10 and 14 months and at 3 years per Group. At 10 months, the NF1 group had lower composite Motor scores (F(1,4) = 7.77, *p* < 0.001, continuous combined z-score of Vineland and Mullen Motor Skills) compared to all other Groups and lower Adaptive function scores (F(1,4) = 3.72, *p* = 0.007, Vineland) compared to EL-ADHD and EL-autism + ADHD. At 3 years, the NF1 group had higher CBCL ADHD scores (F(1,4) = 3.23, *p* = 0.015) compared to the TL group and lower Mullen ELC scores (F(1,4) = 28.04, *p* < 0.001) compared to all other groups. The TL group also had higher Mullen ELC scores compared to the EL-autism and EL-autism + ADHD groups (F(1,4) = 28.04, *p* < 0.001). Considering the Group differences in Motor Skills, all analyses, including Movement were repeated and Motor Skills was included as a control variable.

**Table 1:** Participants characteristics by group

	[1] EL-Autism (*n =* 70)			[2] EL-ADHD (*n =* 28)			[3] EL-Autism + ADHD (*n =* 18)			[4] NF1 (*n =* 29)			[5] Typical Likelihood (*n =* 26)				
	Mean	(SD)	n	Mean	(SD)	n	Mean	(SD)	n	Mean	(SD)	n	Mean	(SD)	n	F(p)	Sig Diff
*10 months*			55			21			13			21			20		
Sex (m:f)^†^	30:25		55	11:10		21	9:4		13	11:10		21	10:10		20	1.38 (.848)	
Age in days	320.33	(14.46)	55	325.95	(23.95)	21	317.15	(11.08)	13	325.81	(17.30)	21	324.00	(16.52)	20	1.02 (.400)	
Motor Skills	0.05	(0.60)	46	0.28	(0.89)	19	0.19	(0.71)	11	−0.67	(0.62)	21	0.51	(0.75)	14	7.77 (<.001)	4 < all
Vineland ABC	91.27	(13.56)	44	99.25	(14.78)	16	98.50	(11.92)	10	85.16	(12.44)	19	98.00	(10.73)	13	3.72 (.007)	4 < 2 & 3
*14 months*			56			20			16			21			18		
Sex (m:f)^†^	26:30		56	14:6		20	10:6		16	9:12		21	11:7		18	5.21 (.266)	
Age in days	448.82	(18.05)	56	449.05	(22.61)	19	452.19	(20.44)	16	454.33	(23.48)	21	449.28	(19.59)	18	0.33 (.855)	
Motor Skills	0.11	(0.73)	52	0.25	(0.69)	14	−0.17	(0.60)	13	−0.37	(0.69)	19	0.00	(0.65)	15	2.28 (.065)	
Vineland ABC	92.48	(11.19)	50	100.71	(13.26)	14	93.08	(12.57)	12	92.61	(9.39)	18	93.86	(9.91)	14	1.57 (.188)	
*3 years*			53			19			13			25			20		
CBCL ADHD	4.51	(3.29)	53	4.74	(3.28)	19	5.69	(4.07)	13	6.28	(2.79)	25	3.10	(2.20)	20	3.23 (.015)	4 > 5
Vineland ABC	94.07	(12.15)	45	96.25	(13.07)	16	89.44	(16.33)	9	92.44	(14.90)	25	101.18	(8.94)	11	1.29 (.280)	
MSEL ELC	105.75	(18.73)	48	118.50	(20.96)	18	104.85	(21.02)	13	74.57	(13.47)	23	129.53	(11.62)	17	28.04 (<.001)	4 < all 1, 3 & 4 < 5

Note: Significant group differences compared using Tukey–Kramer are denoted in the last column “Sig Diff” based on the assigned groups numbers; Motor Skills: combined score from Motor subscales of MSEL and Vineland; EL: Elevated Likelihood; CBCL ADHD: Child Behaviour Checklist – DSM ADHD total score; Vineland ABC: Vineland-II Adaptive Behavior Composite; MSEL ELC: MSEL – Early Learning Composite

^†^Tested using chi-square

### Position

Because of differences in position between children (Lap: 10 m *n =* 29, 14 m: *n =* 38; Highchair: 10 m *n =* 101, 14 m *n =* 93), we looked at the effect of Position on Attention and Movement as well as the interaction of Position with Condition and Group (SM10). We found no main or interaction effects of Position on Attention. There was a main effect of Position on Movement (*p* < 0.001), such that infants sitting on their parent’s lap moved more. There was no interaction with Condition, however we did observe an interaction effect with Group, showing that the EL-autism (*t* = 3.39, *p* = 0.008) and the EL-ADHD groups (*t* = 3.61, *p* = 0.004) showed significantly more Movement (after Bonferroni correction) when sitting on the parent’s lap versus in the high-chair compared to the TL group. None of the other contrasts were significant. Considering the association between Position and Movement and the differences between Groups, we repeated all mixed models that include Movement with Motor Skills and Position as control variables.

**Fig. 3:**
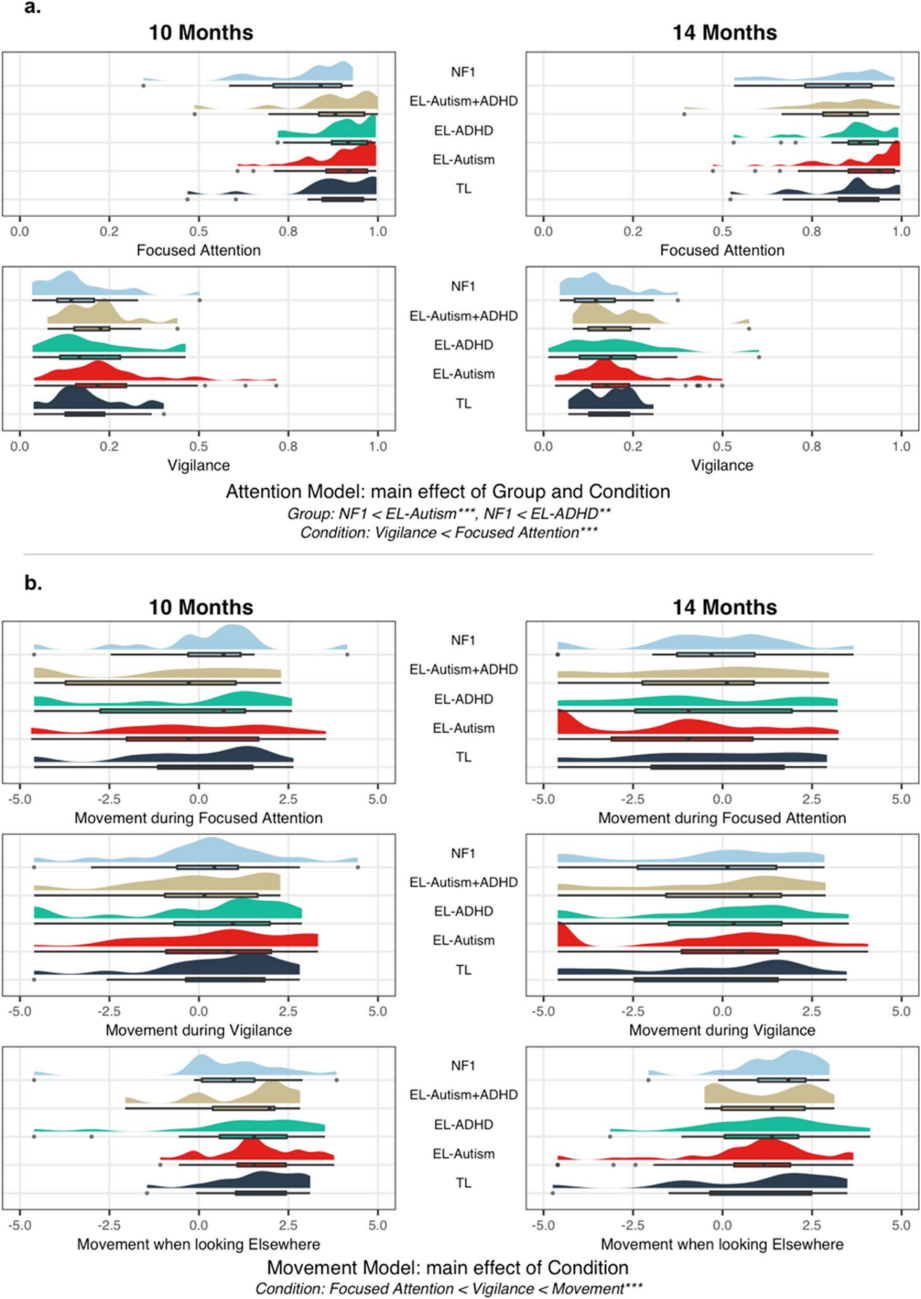
Distributions of **a** Attention and **b** Movement, by Condition, Group and Timepoint. Significant model effects are provided in figure (** *p* <.05, *** *p* <.001)

Figure 3 includes the range and distributions of the untransformed Attention and transformed Movement data (see also SM6 for means, standard deviations and ranges of transformed and untransformed data).

### Attention

Results from the GLM beta regression model (Table 2; Fig. 3) showed a significant effect of Condition (Focused Attention versus Vigilance, *p* < 0.001), and a significant effect of Group (*p* < 0.001). There were no significant effects of Timepoint, Group-by-Condition or Group-by-Condition-by-Timepoint interactions on Attention behaviours. As there was no effect of Timepoint, we did not considered responses at 10 and 14 months separately.

**Table 2:** Model statistics of GLMM model for attention

Effect	Estimate	SE	df	F	*p*	95% CI
Group	−1.70	0.14	4, 170	7.79	<.001***	[−1.98, −1.41]
Condition	3.07	0.23	1, 170	1284.76	<.001***	[2.62, 3.53]
Timepoint	0.08	0.22	1, 170	2.79	0.097	[−0.37, 0.53]
Group x Condition	-	-	4, 170	1.21	0.308	-
Group x Condition x Timepoint	-	-	9, 170	0.33	0.963	-

**Parameter**	**Estimate**	**SE**	**df**	**t**	*p*	**95% CI**
*Groups*						
TL	0.19	0.11	170	1.71	0.089	[−0.03, 0.40]
EL-ADHD	0.33	0.1	170	3.22	<.001***	[0.13, 0.58]
EL-autism	0.46	0.07	170	5.43	<.001***	[0.33, 0.59]
EL-autism + ADHD	0.14	0.12	170	1.20	0.233	[−0.09, 0.38]
NF1	−0.16	−0.16	170	−1.70	0.091	[−0.34, 0.03]
*Conditions*						
Focused Attention	1.79	0.07	170	24.15	<.001***	[1.64, 1.93]
Vigilance	−1.4	0.05	170	−28.37	<.001***	[−1.50, −1.31]

The effect of Condition revealed that across Groups and Timepoints, infants showed more Focused Attention (EMM = 1.79, SE = 0.07) compared to Vigilance (EMM = −1.40, SE = 0.05, OR = 24.39, also see SM11), i.e., they looked more when the puppet was present.

Bonferroni pairwise comparison of Group (across Conditions and Timepoints) revealed that there were no significant differences between the TL, EL-autism, EL-ADHD and EL-autism + ADHD groups (all *p*’s > 0.17, see SM11). The NF1 group (EMM = −0.16, SE = 0.09) showed reduced Attention compared to the EL-autism (EMM = 0.46, SE = 0.07, *t*(1, 170) = 5.43, *p* < 0.001, OR = 1.85) and EL-ADHD groups (EMM = 0.33, SE = 0.10, *t*(1, 170) = 3.53, *p* = 0.005, OR = 1.63), but did not differ from the TL or EL-autism + ADHD groups.

### Movement

The linear mixed model for Movement was significant, Wald ${\chi }^{2}$(29) = 339.52, *p* < 0.001. All model statistics are provided in Table 3 and visualised in Fig. 3. There was a significant main effect of Condition (*p* < 0.001), such that, across Groups and Timepoints, the amount of Movement was lowest during periods of Focused Attention (EMM = −0.57, SE = 0.18), followed by Vigilance (EMM = 0.01, SE = 0.17, 1.65 times higher compared to Focused Attention), and highest when infants were looking Elsewhere (EMM = 1.19, SE = 0.12, 3.46 times higher compared to Vigilance). All contrasts were significant (*p* < 0.001, Table 3). The main effect of Condition accounted for an additional 10.6% of variance explained in the model. No significant main effect of Group, Timepoint or interaction effects were found. Thus, Movement varied based on Condition, but no Group or Timepoint differences were observed.

**Table 3:** Model statistics of GLM for movement

Effect	Estimate	SE	χ2	df	*p*	95% CI
*Movement*						
Group	-	-	0.46	4	.978	-
Condition	-	-	215.25	2	<.001***	-
Timepoint	-	-	1.03	1	.311	-
Group x Condition	-	-	5.80	8	.670	-
Group x Timepoint	-	-	1.68	4	.794	-
Condition x Timepoint	-	-	2.37	2	.306	-
Group x Condition x Timepoint	-	-	8.02	8	.431	-
*Added second model*						-
Position	-	-	23.80	1	<.001***	-
Motor Skills	0.21	0.24	z = −1.10		.383	[-.26, 0.67]

**Parameters**	**Estimate**	**SE**	**z**		***p***	**95% CI**
*Group*						
TL	0.40	0.36	1.09		.284	[−0.31, 1.10]
EL-ADHD	0.24	0.20	1.19		.235	[−0.16, 0.63]
EL-autism	0.12	0.39	0.30		.764	[−0.65, 0.88]
EL-autism + ADHD	0.06	0.46	0.12		.905	[−0.85, 0.96]
NF1	0.24	0.32	0.74		.461	[−0.39, 0.86]
*Condition*						
Focused Attention	1.79	0.07	24.15		<.001***	[1.64, 1.93]
Vigilance	−1.4	0.05	−28.37		<.001***	[−1.50, −1.31]
*Added second model: Position*						
Highchair	−0.16	0.17	−0.93		.354	[−0.49, 0.18]
Lap	1.26	0.24	5.21		<.001	[0.79, 1.74]

**Contrasts**	**Estimate**	**SE**	**z**		***p***	**95% CI**
*Movement*						
Vigilance vs FA	0.59	0.13	4.42		<.001***	[0.27, 0.90]
Elsewhere vs FA	1.77	0.12	14.54		<.001***	[1.48, 2.06]
Elsewhere vs Vigilance	1.18	0.11	11.06		<.001***	[0.93, 1.44]
*Position*						
Lap vs Highchair	1.42	.29	4.88		<.001***	[0.85, 1.99]

**Table 4:** Spearman correlations between Attention/Movement and 3-year traits

	**CBCL ADHD Traits at 3 years**					
	**10 months**			**14 months**		
Attention	*n*	*r*_*s*_	*p*	*n*	*r*_*s*_	*p*
Focused Attention	97	-.27	.008**	102	-.19	.054
Vigilance	97	-.09	.387	102	-.08	.437
Movement						
Focused Attention	75	.07	.551	85	.10	.349
Vigilance	75	-.12	.309	85	.03	.754
Elsewhere	75	-.005	.967	85	.06	.589

Associations were run on non-transformed data using Spearman correlation

Including Position and Motor Skills as control variables did not change the overall model. Position was a significant predictor of Movement (*p* < 0.001; see Table 3), with more Movement observed when the infant sat on the parent’s lap compared to the highchair (*p* < 0.001). There was no effect of Motor Skills on Movement behaviours. To further explore the effect of Position on Movement, we ran the mixed model separately for each Position (see SM12). The results were largely the same, however amount of Movement did not differ between Focused Attention and Vigilance when sitting on the parent’s lap (see also Figure S12).

Inspection of the Q-Q plots showed deviations at the lower end of the plot and deviations on both ends of the BLUP plots. Removing two outliers (standardized residual |> 3|) did not change the model results and did not improve the model fit. Considering the deviations, we repeated the analysis to check the robustness using non-parametric Kruskal–Wallis tests for Condition and Group separately, which showed a similar significant Condition effect (*p* < 0.001), and again no Group effect (*p* = 0.906; see SM12).

### Longitudinal associations with ADHD traits at 3 years

#### Bivariate

**Fig. 4:**
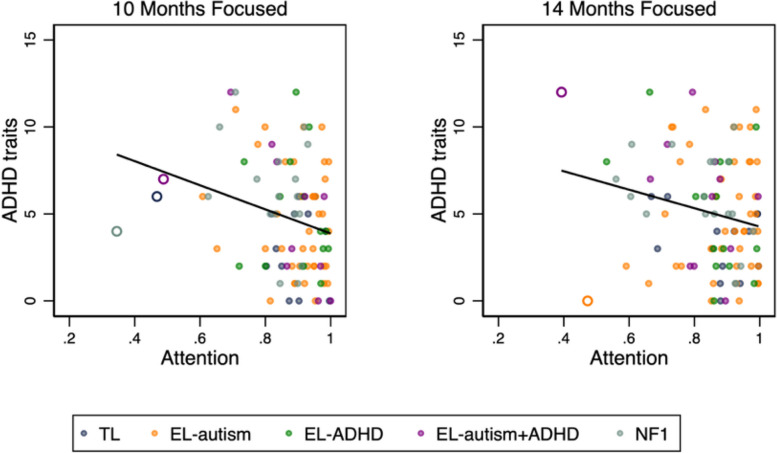
Bivariate associations between Focused Attention at 10 and 14 months and ADHD traits at age 3

First, we ran bi-variate Spearman correlations to look at the association of Attention and Movement with ADHD for 10 and 14 months separately, including all groups (see Table 4). There was a weak significant negative association between 10 months Focused Attention and 3-year ADHD traits (*r*_*s*_ = −0.27*, p* = 0.008; adjusted for four Attention associations:* p* = 0.032). None of the other associations were significant. Figure 4 shows the associations between Focused Attention and CBCL traits at 10 and 14 months (see SM13 for scatterplots of the other associations).

#### Attention-by-movement interaction

**Fig. 5:**
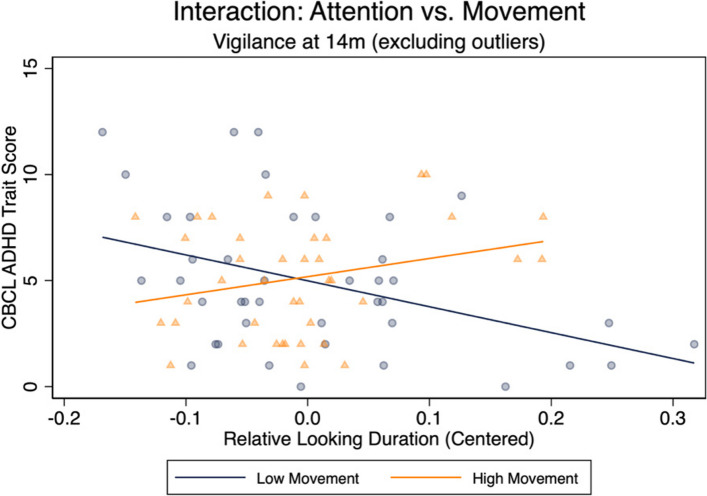
Association of Attention and ADHD traits for infants showing low vs high Movement (median split for visualisation) during Vigilance at 14 months. Figure excludes 4 outliers with high Cook’s d values

Only the Negative Binomial mixed-effect model for Vigilance at 14 months was significant (Wald ${\chi }^{2}$(3) = 20.77, *p* < 0.001, Table S13.1). There were no main effects of Attention or Movement but there was an interaction effect (IRR = 2.13, *p* < 0.001, Fig. 5). Probing the interaction showed that there was a significant association between higher Attention and lower ADHD traits for low movers (−1 SD: marginal effect = −8.00, SE = 3.23, *p* = 0.013) but not high movers (+ 1 SD: marginal effect = −0.48, SE = 3.85, *p* = 0.900). Removing four datapoints with high Cook’s *d* values did not change the results. Adding Motor Skills and Position did also not change the results and neither was a significant predictor (see SM13, Table S13.2).

## Discussion

Here we investigated early precursors of ADHD traits in infants with a family history or monogenic condition associated with elevated rates of ADHD. We specifically examined measures of Focused Attention, Vigilance and Movement during the different attention states to capture both activity and attention-related components of the ADHD phenotype. Specifically, we followed infants at 10 and 14 months who – either through a family history of neurodevelopmental conditions or a diagnosis of NF1 – have an elevated likelihood of developing ADHD traits. By using a puppet task that combined behavioural coding with accelerometer data, we investigated early differences in attention and movement between groups with an elevated and typical ADHD likelihood and assessed their associations with three-year ADHD traits. We did not find a main or interaction effect of Timepoint in either model, suggesting attention and movement patterns were similar at 10 and 14 months. Contrary to our expectations, we did not observe reduced Attention in the EL-autism, EL-ADHD or EL-autism + ADHD groups compared to the TL group; only the NF1 group showed lower levels of Attention across Focused Attention and Vigilance conditions (specifically compared to the EL-autism and EL-ADHD groups). Furthermore, infant Activity levels did not differ between Groups or Timepoints, but differed by Condition (lowest in Focused Attention, highest when Looking Elsewhere). In relation to later traits, we found that greater Focused Attention at 10 months was associated with fewer ADHD traits at age three. Finally, increased Vigilance at 14 months predicted fewer ADHD traits, but this was moderated by Movement: the effect of Attention was primarily observed in infants also showing lower Activity levels. In summary, these results suggest that Focused Attention and Attention-Movement coupling may emerge as early signs of attention differences in infants with an elevated likelihood for ADHD traits over the first years of life.

### Reduced focused and vigilant attention in NF1

Our findings demonstrate that, at 10 and 14 months, NF1 infants showed less Focused Attention and Vigilance compared to the EL-autism and EL-ADHD group, but not the TL or EL-Autism + ADHD groups. However, the direction of effects was the same for the latter two Groups. These had slightly smaller sample sizes and thus in part it is possible that the lack of a significant difference is related to lack of power. However, the group with NF1 did show significantly lower Attention than the EL-ADHD group, which was of a comparable size. All infants in the NF1 group had a diagnosed monogenic condition, whereas infants in the EL-ADHD group likely have heterogenous polygenic profiles; thus, it may be that differences in attention are more consistent across the NF1 group. Our study is one of the first to show that reduced Focused Attention and Vigilance persisted across 10 and 14 months in infants with NF1 in comparison to other likelihood groups; few studies have examined the early cognitive development of NF1 longitudinally [49, 50]. Our results are broadly consistent with a second report from the same cohort, in which infants showed slower progression in attentional behaviours between 10 and 14 months during two eye-tracking tasks compared to the TL infants and EL-ADHD [24]. Taken together, these studies suggest that early fragilities in attention maybe be observed from infancy in NF1. Further work in larger cohorts is required to determine whether these changes precede or contribute to the emergence of other behavioural and cognitive difficulties in NF1, since alterations in attention could affect a child’s ability to focus and benefit from learning opportunities at home and at school. For example, previous cross-sectional studies have reported lower cognitive abilities in preschool aged NF1 children [51] and difficulties and delays in many aspects of development, including attention and executive functions in older children [52–54], but future research is needed to better characterise differences in developmental trajectories from infancy to childhood in this population.

### Objectively measured activity levels did not differ across groups

In the present study, infants showed the least Movement during Focused Attention, more Movement during Vigilance and the most when looking elsewhere, showing that infants already modulate their movement in relation to their attention state. Contrary to our predictions, we found no differences in the amount of Movement between EL and TL infants at both 10 and 14 months, despite group differences in observed and reported motor skills, with the NF1 group having a significantly lower composite score. Consistent with our findings, we have previously reported no differences in activity levels among 10-month-old infants with and without a family history of autism and/or ADHD, using parent reports, experimenter ratings, and objective measures of head motion [25]. However, other studies have found higher activity levels in infants with EL-ADHD [26, 31]. Using a latent profile analysis, Miller et al., (2020) reported a higher increase in “out of seats behaviours” in infants with EL-ADHD from 12 to 24 months compared to TL infants with differences remaining significant at 36 months [31]. Furthermore, Reetzke et al., (2022) described a heightened level of motor activity—indexed by accelerometer—at 18 months in infants with EL-ADHD and EL-autism [26]. These differences could be due to the variability in the settings in which behaviours were assessed across studies. For example, in our experimental live puppet task infants were seated either on parents' laps or in highchair during the puppet events, which may have restricted their ability to move. Indeed, in our study movement was more reduced when infants were seated in a more restrictive highchair compared to the parent’s lap. In contrast, previous studies measured children’s activity levels during tabletop tasks when children are generally free to move [26, 31]. Thus, early differences in activity level in ADHD may be more readily captured in free moving contexts. It is also important to note that detecting differences in movement within the first year of life is challenging due to infants’ limited locomotor abilities and differences may become more apparent as children begin to walk independently. Consequently, differences in these behaviours may be more readily detected during the second year of life, as reported in previous studies [26, 31]. To understand the timing of emerging differences in behaviours, future research should focus on implementing a variety of methods in different contexts that allow for capturing behaviours more broadly.

### Focused attention predicted later ADHD traits

We did not observe differences in attention at 10 and 14 months between infants with and without a family history of ADHD. However, reduced Focused Attention at 10 months was associated with later ADHD traits. This is consistent with some other studies; for example, Lawson & Ruff described that infants’ measure of focused attention to an object at 7 months predicted infants’ hyperactivity and impulsivity behaviours at 4/5 years of age [27]. Other studies have reported more inattentive behaviours in EL-ADHD compared to TL infants [18, 31]; it is likely that the variation in the number of children with later ADHD within a cohort of EL-ADHD infants influences the detection of family history group differences. Differences in the sensitivity of different measures of attention to ADHD effects may also relate to methodological variations in how attention behaviours were measured. For example, in Miller et al. (2021) attention was evaluated both by researchers’ observations and through behavioural coding during a developmental assessment and several experimental tasks [18]. Reports from researchers' ratings of inattentive behaviours were higher for infants with EL-ADHD compared to the TL infants, but coded attention did not differ between groups at either 12, 18, or 24 months, which is consistent with our findings. Observer reports may offer a broader view of infants’ behaviours across various settings, but these methods may be subject to bias [55] (i.e. parental/researchers report of infant behaviour may be subjectively influenced by their experience with other children, potentially leading to under- or over-reporting of certain behaviours). Similarly, researchers’ awareness of familial likelihood status can shape their interpretation of observed behaviours. Observational methods during structured tasks (detailed video coding), as used in the current study and in Miller et al. (2021) study, can provide a more objective behavioural analysis, minimizing measurement bias [18, 39]. However, such tasks like our puppet task often involve the infant sitting down with tasks that are designed to elicit their attention. In line with this, we also found no differences at 10 months using eye-tracking to measure infants’ attention in our family history sample [25]. It may be that tasks that require the child to control their own attention more effectively are more sensitive to the presence of emerging ADHD than tasks with strong attentional cues.

Alternatively, emerging group differences may not be readily observable during the first year of life but may become more distinguishable during the latter half of the second year. ADHD is typically not diagnosed until school age or older, and it is likely therefore that clear behavioural differences emerge slowly over developmental time. Additionally, capturing subtle early differences presents a methodological challenge, particularly given that only a small proportion of infants in the sample are likely to be on a developmental pathway toward ADHD or autism. Within the familial likelihood groups, only a proportion of the infants will later be diagnosed with ADHD and many will develop typically. In contrast, the NF1 group all have a diagnosed genetic condition, which may enable earlier detection of behavioural differences at a group level due to more pronounced and consistent behavioural profiles. In line with this, the EL autism and ADHD groups showed fewer differences to the typical likelihood group on other developmental measures. The NF1 group, in contrast, had the lowest adaptive behaviour scores at 10 months and the lowest developmental and highest ADHD scores at 3 years, suggesting that this group was the most atypical overall. It is possible that ADHD associated with polygenic factors has a different early developmental profile than ADHD associated with monogenic conditions; future studies with larger samples and diagnostic classification in mid-childhood would be required to test this possibility.

We did not observe associations between Vigilance and later ADHD traits at 10 months. Focused Attention and Vigilance likely do not emerge concurrently within the first year of life. These two endogenous aspects of attention may follow distinct developmental trajectories and contribute to different cognitive functions across development. Focused Attention emerges during the second half of the first year of life and has been linked to broader developmental outcomes, including executive functions, self-regulation, and learning [56, 57]. In contrast, Vigilance may develop later, during the second year of life, and rely more heavily on cognitive resources and task demands in older children [58]. Indeed, we did find a Vigilance-by-Movement interaction effect at 14 months. We predicted that better Attention (Focused Attention and Vigilance) combined with less Movement would be associated with a lower level of ADHD at 3 years. Our findings are broadly consistent with this prediction but are specific to Vigilance. More Vigilance was associated with fewer ADHD traits, but only for those children showing lower versus higher levels of Movement. In other words, the interaction suggests that a mismatch between Attention state (here, during Vigilance) and Movement could be particularly important in relation to later ADHD traits. Our findings also show that, across the sample, infants show a coupling between their Attention state and amount of Movement, and this coupling may be an important factor to consider when evaluating attentive states in infants. Future research should, however, consider whether attention-movement coupling is impacted by any emerging differences in motor abilities as children become more mobile.

In the study conducted by Shephard et al., parents' reports on attention at 7, 10 and 14 months did not show any associations with later ADHD traits, and only inhibitory control at 24 months was associated with ADHD symptoms of inattention and activity levels [59]. Our observation of altered coupling between Looking and Movement may reflect reduced inhibitory control in emerging ADHD. Examining the link between movement during attention and measures of inhibitory control could further elucidate this association. Further, it will be important to determine whether motion during looking is distracting or adaptive for the infant. Movement may be necessary to help focus infants with emerging ADHD, which would have important implications for how it is considered in treatment programmes.

### Strengths and limitations

This study is among the first to explore the emerging behavioural features of ADHD traits in a diverse cohort of infants with an EL of ADHD due to familial history and infants with NF1. Implementing longitudinal prospective studies of infants with NF1 within the ‘infant sibling’ design is a valuable approach to understanding the early features of ADHD and the effects on phenotypic variabilities of symptoms across co-occurring conditions. In our study, we also employed an infant-tailored experimental paradigm that enabled us to simultaneously assess Attention and Movement, allowing us to more directly compare their predictive validity for ADHD. We examined two components of attention, Focused attention and Vigilance, that characterise ADHD profiles [32, 34]. Vigilance has often been measured in older children and adults using computerised tasks, but has rarely been applied in infant studies. We also assessed body movements using an accelerometery that unlike parents/experimenter reports offers an objective behavioural measure. Although we report a lack of differences in movement at this early age, other studies using accelerometer measures distinguished preschool children with EL-ADHD from TL children [60, 61] showing that this methodological approach is feasible for studying behaviours in early development and has the potential to uncover the developmental pathways of motor behaviours in these cohorts of infants and to further identify the emergence of these characteristics and their impact on children’s outcomes.

Another strength of our study is the high retention rate, particularly up to 3 years (76% of children seen at 10 and/or 14 months). These retention rates are strong for longitudinal infant research and enhance the robustness of the findings by reducing attrition bias and preserving statistical power. It should be noted however that the overlap of children who had data available at both infant timepoints is lower (53% for looking behaviour, 28% for movement), which was mainly due to technical issues.

The present study has some limitations. With regards to the Movement data, some children were sitting on their parent’s lap, which may mean some of the movements that are being picked up are from the parent and/or that children were less restricted in their movements. Further, we do not know how Vigilance measured during the puppet task in infants correlates with Vigilance and other aspects of Sustained Attention in later childhood. Using a computerized Vigilance paradigm based on the puppet task (the Early Childhood Vigilance Task; ECVT), Goldman et al. [62] reported a moderate relation between vigilance and sustained attention during free play in 2-year-olds, although no relationship was found with parental ratings of attention. Therefore, more research is needed to determine how early task-based vigilance relates to infants' attentional development and whether it provides a reliable early marker of later ADHD-related difficulties.

We examined attention and movements at 10 and 14 months only. Follow-up visits are necessary to assess patterns of developmental trajectories across different behavioural domains considering the variability in the emergence of behaviours between and within infants with an elevated likelihood of developing ADHD. A few longitudinal studies have showed that differences in attention and activity level may emerge later in the second year of life [31]. Equally, we measured ADHD traits at age 3 years, and only a small proportion of children scored in the (borderline) clinical range of the CBCL. Although traits may be relatively stable in some children from toddlerhood, they are likely still emerging, and a diagnosis of ADHD is usually given later in childhood [63]. Here, we found a weak association between ADHD and Attention, which may become more pronounced over time. It would be of interest to understand whether associations between movement, attention and attention-movement coupling with later traits differ by ADHD subtype. For example, less attention coupled with increased movement may be more observed in the hyperactive/impulse subtype compared to the inattentive subtype. Further, because of the skewness and range of our data (proportion data for Attention and positively skewed data for Movement), some of the models we intended to run did not converge or fit well. This meant we used both transformed and untransformed data and decided to confirm our analyses using less parsimonious, non-parametric models. These models were used to confirm the robustness of our model results. Future investigations should also endeavour to follow up these cohorts of children in mid-childhood to distinguish children who meet diagnostic outcomes, as well as to identify which early features might contribute to a later diagnosis.

## Conclusion

In conclusion, our study identified reduced focused attention and vigilance in infants with NF1, but not those with a familial likelihood of ADHD. Across groups and ages, infants showed fewer movements during periods of focused Attention and vigilance, indicating the presence of robust attention-movement coupling. Further, reduced Focused Attention at 10 months and the coupling of Vigilance and Movement at 14 months were associated with ADHD traits at 3 years. Our findings suggest that across the first and early second years of life, attentional differences are evident in infants with NF1. This may indicate the value of intervention programmes aimed at supporting early attentional development in NF1 and highlights the importance of considering early emerging behavioural differences in this genetic syndrome so that support can be provided before children encounter substantial challenges at school. The associations found between Focused Attention at 10 months, as well as the relationship between Vigilance and Movement at 14 months and ADHD-related traits might indicate that these two aspects of attention might emerge at different stages of development and contribute to distinct aspects of later behaviours in childhood. Taken together, our results indicate the importance of considering early attention as a potential domain of vulnerability in infants with emerging ADHD.

## Supplementary Information

Supplementary Material 1.

## Data Availability

The datasets generated and/or analysed during the current study are not publicly available due to confidentiality constraints within our ethical approvals. However, access may be granted upon completion of a successful Project Affiliation Form via The BASIS/STAARS Network ([http://www.basisnetwork.org/]) and completion of all requisite data access and sharing protocols. Please get in touch with the corresponding authors to start this process.

## Abbreviations

EL: Elevated Likelihood
ADHD: Attention Deficit Hyperactivity Disorder
BLUP: Best Linear Unbiased Predictors
CBCL: Child Behaviour Checklist
DSM: Diagnostic and Statistical Manual of Mental Disorders
EDEN: Early Development in Neurofibromatosis Type 1
GLMM: Generalized Linear Mixed-effects Model
MSEL-ELC: Mullen Scales of Early Learning – Early Learning Composite
NF1: Neurofibromatosis
SM: Supplemental Materials
STAARS: Studying Autism and ADHD in the eaRly yearS
VABS-ABC: Vineland Adaptive Behavior Scales – Adaptive Behaviour Composite

## References

[1] American Psychiatric Association. Diagnostic and statistical manual of mental disorders: DSM-5. 5th edition. Arlington,VA: Author; 2013.

[2] Polanczyk G, De Lima MS, Horta BL, Biederman J, Rohde LA. The worldwide prevalence of ADHD: a systematic review and metaregression analysis. Am J Psychiatry. 2007;164:942–8. 10.1176/ajp.2007.164.6.942. 17541055 10.1176/ajp.2007.164.6.942

[3] Polanczyk GV, Salum GA, Sugaya LS, Caye A, Rohde LA. Annual research review: a meta‐analysis of the worldwide prevalence of mental disorders in children and adolescents. J Child Psychol Psychiatry. 2015;56:345–65. 10.1111/jcpp.12381. 25649325 10.1111/jcpp.12381

[4] Polanczyk G, Caspi A, Houts R, Kollins SH, Rohde LA, Moffitt TE. Implications of extending the ADHD age-of-onset criterion to age 12: results from a prospectively studied birth cohort. J Am Acad Child Adolesc Psychiatry. 2010;49:210–6. 10.1016/j.jaac.2009.12.014. 20410710

[5] Bufferd SJ, Dougherty LR, Carlson GA, Rose S, Klein DN. Psychiatric disorders in preschoolers: continuity from ages 3 to 6. Am J Psychiatry. 2012;169:1157–64. 10.1176/appi.ajp.2012.12020268. 23128922 10.1176/appi.ajp.2012.12020268 PMC3513401

[6] Meeuwsen M, Perra O, Van Goozen SHM, Hay DF. Informants’ ratings of activity level in infancy predict ADHD symptoms and diagnoses in childhood. Dev Psychopathol. 2019;31:1255–69. 10.1017/S0954579418000597. 30319083 10.1017/S0954579418000597

[7] Wichstrøm L, Belsky J, Steinsbekk S. Homotypic and heterotypic continuity of symptoms of psychiatric disorders from age 4 to 10 years: a dynamic panel model. J Child Psychol Psychiatry. 2017;58:1239–47. 10.1111/jcpp.12754. 28543077 10.1111/jcpp.12754

[8] Danckaerts M, Sonuga-Barke EJS, Banaschewski T, Buitelaar J, Döpfner M, Hollis C, et al. The quality of life of children with attention deficit/hyperactivity disorder: a systematic review. Eur Child Adolesc Psychiatry. 2010;19:83–105. 10.1007/s00787-009-0046-3. 19633992 10.1007/s00787-009-0046-3 PMC3128746

[9] Visser SN, Danielson ML, Bitsko RH, Holbrook JR, Kogan MD, Ghandour RM, et al. Trends in the parent-report of health care provider-diagnosed and medicated attention-deficit/hyperactivity disorder: United States, 2003-2011. J Am Acad Child Adolesc Psychiatry. 2014;53. 10.1016/j.jaac.2013.09.001. 10.1016/j.jaac.2013.09.001 PMC4473855 24342384

[10] Sonuga-Barke EJS, Halperin JM. Developmental phenotypes and causal pathways in attention deficit hyperactivity disorder: potential targets for early intervention? J Child Psychol Psychiatry. 2010;51:368–89. 10.1111/j.1469-7610.2009.02195.x. 20015192 10.1111/j.1469-7610.2009.02195.x

[11] Faraone SV, Larsson H. Genetics of attention deficit hyperactivity disorder. Mol Psychiatry. 2019;24:562–75. 10.1038/s41380-018-0070-0. 29892054 10.1038/s41380-018-0070-0 PMC6477889

[12] Miller M, Musser ED, Young GS, Olson B, Steiner RD, Nigg JT. Sibling recurrence risk and cross-aggregation of attention-deficit/hyperactivity disorder and autism spectrum disorder. JAMA Pediatr. 2019;173:147. 10.1001/jamapediatrics.2018.4076. 30535156 10.1001/jamapediatrics.2018.4076 PMC6439602

[13] Musser ED, Hawkey E, Kachan‐Liu SS, Lees P, Roullet J, Goddard K, et al. Shared familial transmission of autism spectrum and attention‐deficit/hyperactivity disorders. J Child Psychol Psychiatry. 2014;55:819–27. 10.1111/jcpp.12201. 24444366 10.1111/jcpp.12201 PMC4211282

[14] Bussu G, Jones EJH, Charman T, Johnson MH, Buitelaar JK, BASIS Team. Latent trajectories of adaptive behaviour in infants at high and low familial risk for autism spectrum disorder. Mol Autism. x2019;10:13. 10.1186/s13229-019-0264-6. 30923608 10.1186/s13229-019-0264-6 PMC6420730

[15] Szatmari P, Chawarska K, Dawson G, Georgiades S, Landa R, Lord C, et al. Prospective longitudinal studies of infant siblings of children with autism: lessons learned and future directions. J Am Acad Child Adolesc Psychiatry. 2016;55:179–87. 10.1016/J.JAAC.2015.12.014. 26903251 10.1016/j.jaac.2015.12.014 PMC4871151

[16] Begum Ali J, Charman T, Johnson MH, Jones EJH, the BASIS/STAARS Team. Early motor differences in infants at elevated likelihood of autism spectrum disorder and/or attention deficit hyperactivity disorder. J Autism Dev Disord. 2020;50:4367–84. 10.1007/s10803-020-04489-1. 32328858 10.1007/s10803-020-04489-1 PMC7677154

[17] Gui A, Mason L, Gliga T, Hendry A, Begum Ali J, Pasco G, et al. Look duration at the face as a developmental endophenotype: elucidating pathways to autism and ADHD. Dev Psychopathol. 2020;32:1303–22. 10.1017/S0954579420000930. 33012299 10.1017/S0954579420000930

[18] Miller M, Iosif A-M, Bell LJ, Farquhar-Leicester A, Hatch B, Hill A, et al. Can familial risk for ADHD be detected in the first two years of life? J Clin Child Adolesc Psychol. 2021;50:619–31. 10.1080/15374416.2019.1709196. 31951755 10.1080/15374416.2019.1709196 PMC7365744

[19] Lo-Castro A, D’Agati E, Curatolo P. ADHD and genetic syndromes. Brain Dev. 2011;33:456–61. 10.1016/j.braindev.2010.05.011. 20573461 10.1016/j.braindev.2010.05.011

[20] Evans D, Howard E, Giblin C, Clancy T, Spencer H, Huson S, et al. Birth incidence and prevalence of tumor-prone syndromes: estimates from a UK family genetic register service. Am J Med Genet A. 2010;152A:327–32. 10.1002/ajmg.a.33139. 20082463 10.1002/ajmg.a.33139

[21] Uusitalo E, Leppävirta J, Koffert A, Suominen S, Vahtera J, Vahlberg T, et al. Incidence and mortality of Neurofibromatosis: a total population study in Finland. J Invest Dermatol. 2015;135:904–6. 10.1038/jid.2014.465. 25354145 10.1038/jid.2014.465

[22] Kayl AE, Moore BD. Behavioral phenotype of Neurofibromatosis, Type 1. Ment Retard Dev Disabil Res Rev. 2000;6:117–24. 10.1002/1098-2779(2000)6:2/3C117::AID-MRDD5/3E3.0.CO;2-X. 10899804 10.1002/1098-2779(2000)6:2<117::AID-MRDD5>3.0.CO;2-X

[23] Garg S, Lehtonen A, Huson SM, Emsley R, Trump D, Evans DG, et al. Autism and other psychiatric comorbidity in Neurofibromatosis Type 1: evidence from a population‐based study. Dev Med Child Neurol. 2013;55:139–45. 10.1111/dmcn.12043. 23163236 10.1111/dmcn.12043

[24] Begum Ali J, Mason L, Charman T, Johnson MH, Green J, Garg S, et al. Disrupted visual attention relates to cognitive development in infants with Neurofibromatosis Type 1. J Neurodev Disord. 2025;17:12. 10.1186/s11689-025-09599-4. 40087579 10.1186/s11689-025-09599-4 PMC11907931

[25] Goodwin A, Hendry A, Mason L, Bazelmans T, Begum Ali J, Pasco G, et al. Behavioural measures of infant activity but not attention associate with later preschool adhd traits. Brain Sci. 2021;11. 10.3390/brainsci11050524. 10.3390/brainsci11050524 PMC8143002 33919004

[26] Reetzke R, Iosif A, Hatch B, De La Paz L, Chuang A, Ozonoff S, et al. Patterns of objectively measured motor activity among infants developing ASD and concerns for ADHD. J Child Psychol Psychiatry. 2022;63:663–73. 10.1111/jcpp.13504. 34387359 10.1111/jcpp.13504 PMC8841001

[27] Lawson KR, Ruff HA. Early focused attention predicts outcome for children born prematurely. 2004;25. 10.1097/00004703-200412000-00003. 15613988 10.1097/00004703-200412000-00003

[28] Ruff HA, Lawson KR, Parrinello R. Long-term stability of individual differences in sustained attention in the early years. 1990;61. 10.2307/1131047. 2307047

[29] Colombo J. The development of visual attention in infancy. Annu Rev Psychol. 2001;52:337–67. 10.1146/annurev.psych.52.1.337. 11148309 10.1146/annurev.psych.52.1.337

[30] Auerbach JG, Berger A, Atzaba-Poria N, Arbelle S, Cypin N, Friedman A, et al. Temperament at 7, 12, and 25 months in children at familial risk for ADHD. Infant Child Dev. 2008;17:321–38. 10.1002/icd.579.

[31] Miller M, Austin S, Iosif AM, De La Paz L, Chuang A, Hatch B, et al. Shared and distinct developmental pathways to ASD and ADHD phenotypes among infants at familial risk. Dev Psychopathol. 2020;32:1323–34. 10.1017/S0954579420000735. 32933597 10.1017/S0954579420000735 PMC7891894

[32] Huang-Pollock CL, Karalunas SL, Tam H, Moore AN. Evaluating vigilance deficits in ADHD: a meta-analysis of CPT performance. J Abnorm Psychol. 2012;121:360–71. 10.1037/a0027205. 22428793 10.1037/a0027205 PMC3664643

[33] Leung J-P, Leung PWL, Tang CSK. A vigilance study of ADHD and control children: event rate and extra-task stimulation. 2000;12. 10.1023/A:1009409720485.

[34] Tucha L, Tucha O, Walitza S, Sontag TA, Laufktter R, Linder M, et al. Vigilance and sustained attention in children and adults with ADHD. J Atten Disord. 2009;12:410–21. 10.1177/1087054708315065. 18400983 10.1177/1087054708315065

[35] Ruff HA, Capozzoli M, Dubiner K, Parrinello R. A measure of vigilance in infancy. Infant Behav Dev. 1990;13:1–20. 10.1016/0163-6383(90)90002-P.

[36] Hendry A, Johnson MH, Holmboe K. Early development of visual attention: change, stability, and longitudinal associations. Annu Rev Dev Psychol. 2019;1:251–75. 10.1146/annurev-devpsych-121318-085114.

[37] Conners CK, Goldstein S. Conners early childhood: manual. Toronto, ON: Multi-Health Systems Incorporated; 2009.

[38] Conners CK. Conners 3rd Edition Manual. New York: Multi-Health Systems; 2008.

[39] Miller M, Iosif AM, Young GS, Hill MM, Ozonoff S. Early detection of ADHD: insights from infant siblings of children with Autism. J Clin Child Adolesc Psychol. 2018;47:737–44. 10.1080/15374416.2016.1220314. 27732091 10.1080/15374416.2016.1220314 PMC5436956

[40] Littler M, Morton NE. Segregation analysis of peripheral Neurofibromatosis (NF1). J Med Genet. 1990;27:307–10. 10.1136/jmg.27.5.307. 2112607 10.1136/jmg.27.5.307 PMC1017081

[41] Garg S, Plasschaert E, Descheemaeker M-J, Huson S, Borghgraef M, Vogels A, et al. Autism spectrum disorder profile in Neurofibromatosis Type I. J Autism Dev Disord. 2015;45:1649–57. 10.1007/s10803-014-2321-5. 25475362 10.1007/s10803-014-2321-5

[42] National Institutes of Health. Neurofibromatosis: National Institutes of Health consensus development coference statement. Arch Neurol. 1988;45:575–8. 3128965

[43] Mullen E. Mullen scales of early learning. American Guidance Service; 1995.

[44] Sparrow S, Cicchetti DV, Balla DA. Vineland adaptive behavior scales. 2nd ed. Circle Pines, MN: American Guidance Service; 2005.

[45] Achenbach TM, Rescorla LA. Manual for the ASEBA preschool and school-age forms and profiles. Burlington, VT: University of Vermont, Department of Psychiatry; 2001.

[46] StataCorp. Stata statistical software: release 19. College Station, TX: StataCorp LLC; 2025.

[47] West BT, Welch KB, Gałecki AT, Gillespie BW. Linear mixed models: a practical guide using statistical software. 3rd ed. Boca Raton: Chapman and Hall/CRC; 2022. 10.1201/9781003181064.

[48] Rights JD, Sterba SK. Quantifying explained variance in multilevel models: an integrative framework for defining R-squared measures. Psychol Methods. 2019;24:309–38. 10.1037/met0000184. 29999378 10.1037/met0000184

[49] Slevin H, Kehinde F, Begum-Ali J, Ellis C, Burkitt-Wright E, Green J, et al. Developmental trajectories in infants and pre-school children with Neurofibromatosis 1. Mol Autism. 2024;15:45. 10.1186/s13229-024-00621-5. 39407332 10.1186/s13229-024-00621-5 PMC11481376

[50] Garg S, Wan MW, Begum-Ali J, Kolesnik-Taylor A, Green J, Johnson MH, et al. Early developmental trajectories in infants with neurofibromatosis 1. Front Psychol. 2022;13. 10.3389/fpsyg.2022.795951. 10.3389/fpsyg.2022.795951 PMC9355323 35936291

[51] Lorenzo J, Barton B, Arnold SS, North KN. Developmental trajectories of young children with Neurofibromatosis Type 1: a longitudinal study from 21 to 40 months of age. J Pediatr. 2015;166:1006-1012.e1. 10.1016/j.jpeds.2014.12.012. 25598303 10.1016/j.jpeds.2014.12.012

[52] Hyman SL, Shores ; Arthur, North KN. The nature and frequency of cognitive deficits in children with neurofibromatosis type 1. 2005. 10.1212/01.wnl.0000179303.72345.ce. 16217056 10.1212/01.wnl.0000179303.72345.ce

[53] Heimgärtner M, Granström S, Haas-Lude K, Leark RA, Mautner V-F, Lidzba K. Attention deficit predicts intellectual functioning in children with Neurofibromatosis Type 1. Int J Pediatr. 2019;2019:1–10. 10.1155/2019/9493837. 10.1155/2019/9493837 PMC6930769 31915440

[54] Lehtonen A, Howie E, Trump D, Huson SM. Behaviour in children with Neurofibromatosis Type 1: cognition, executive function, attention, emotion, and social competence. Dev Med Child Neurol. 2013;55:111–25. 10.1111/j.1469-8749.2012.04399.x. 22934576 10.1111/j.1469-8749.2012.04399.x

[55] Najman JM, Williams GM, Nikles J, Spence S, Bor W, O’Callaghan M, et al. Bias influencing maternal reports of child behaviour and emotional state. Soc Psychiatry Psychiatr Epidemiol. 2001;36:186–94. 10.1007/s001270170062. 11518032 10.1007/s001270170062

[56] Cheng C, Kaldy Z, Blaser E. Focused attention predicts visual working memory performance in 13-month-old infants: a pupillometric study. Dev Cogn Neurosci. 2019;36:100616. 10.1016/j.dcn.2019.100616. 30769261 10.1016/j.dcn.2019.100616 PMC6555424

[57] Hendry A, Jones EJH, Charman T. Executive function in the first three years of life: precursors, predictors and patterns. Dev Rev. 2016;42:1–33. 10.1016/j.dr.2016.06.005.

[58] Golob EJ, Nelson JT, Scheuerman J, Venable KB, Mock JR. Auditory spatial attention gradients and cognitive control as a function of vigilance. Psychophysiology. 2021;58:e13903. 10.1111/psyp.13903. 34342887 10.1111/psyp.13903 PMC8419090

[59] Shephard E, Bedford R, Milosavljevic B, Gliga T, Jones EJH, Pickles A, et al. Early developmental pathways to childhood symptoms of attention‐deficit hyperactivity disorder, anxiety and autism spectrum disorder. J Child Psychol Psychiatry. 2019;60:963–74. 10.1111/jcpp.12947. 29963709 10.1111/jcpp.12947 PMC6694009

[60] De Crescenzo F, Licchelli S, Ciabattini M, Menghini D, Armando M, Alfieri P, et al. The use of actigraphy in the monitoring of sleep and activity in ADHD: a meta-analysis. Sleep Med Rev. 2016;26:9–20. 10.1016/j.smrv.2015.04.002. 26163053 10.1016/j.smrv.2015.04.002

[61] García Murillo L, Cortese S, Anderson D, Di Martino A, Castellanos FX. Locomotor activity measures in the diagnosis of attention deficit hyperactivity disorder: meta-analyses and new findings. J Neurosci Methods. 2015;252:14–26. 10.1016/j.jneumeth.2015.03.001. 25770940 10.1016/j.jneumeth.2015.03.001 PMC4522351

[62] Goldman DZ, Shapiro EG, Nelson CA. Measurement of vigilance in 2-year-old children. Dev Neuropsychol. 2004;25:227–50. 10.1207/s15326942dn2503_1. 15147998 10.1207/s15326942dn2503_1

[63] Hoang U, James AC, Liyanage H, Jones S, Joy M, Blair M, et al. Determinants of inter-practice variation in ADHD diagnosis and stimulant prescribing: cross-sectional database study of a national surveillance network. BMJ Evid-Based Med. 2019;24:155–61. 10.1136/bmjebm-2018-111133. 30765384 10.1136/bmjebm-2018-111133 PMC6678046

