# Supplementary material for: Decreased attention in 10- and 14-month-olds with neurofibromatosis type 1 and association with later ADHD traits
Source: J Neurodev Disord. 2026 May 9;18:40. doi: 10.1186/s11689-026-09702-3 (PMC13330184; doi:10.1186/s11689-026-09702-3)
Supplement: Supplementary file 1 — Supplementary Material 1. [file 11689_2026_9702_MOESM1_ESM.docx]

Supplementary Materials: Decreased attention in 10- and 14-month-olds with neurofibromatosis type 1 and association with later ADHD traits

AUTHORS: Tessel Bazelmans, Francesca Penza, Jannath Begum-Ali, Chloe Taylor, Mark H. Johnson, Tony Charman, Jonathan Green, Shruti Garg, Emily J.H. Jones, the STAARS and EDEN Teams

Table of Contents

[SM1. ADHD characterisation 2](#_Toc226042383)

[SM2. Data availability and Inclusion/Exclusion comparison 3](#_Toc226042384)

[SM3. Comparison of participants with and without data 5](#_Toc226042385)

[SM4. Average duration of puppets across groups 6](#_Toc226042386)

[SM5. Reliability of Attention behaviour coding 6](#_Toc226042387)

[SM6. Summary of Attention and Movement variables (untransformed & transformed) 7](#_Toc226042388)

[SM7. Mullen Scales of Early Learning 9](#_Toc226042389)

[SM8. Correlations of Mullen and Vineland Motor scales 9](#_Toc226042390)

[SM9. Model effects and interactions for Transformed Attention variable using Linear Mixed Model 10](#_Toc226042391)

[SM10. Effect of position 12](#_Toc226042392)

[SM11. Additional information for Attention GLMM Model 15](#_Toc226042393)

[SM12. Additional Data for Movement Model 16](#_Toc226042394)

[SM13. Associations between Attention and Movements and ADHD traits at 3 years. 19](#_Toc226042395)

[References 22](#_Toc226042396)

# SM1. ADHD characterisation

| **Table S1** *Diagnosis vs Screened Siblings and Parents with ADHD*  **Groups** | | |
| --- | --- | --- |
|  | **EL-ADHD** | **EL-autism+ADHD** |
| Sibling diagnosis | 7 | 15 |
| Sibling screened | 1 | 1 |
| Parent diagnosis | 18 | 2 |
| Parent screened | 4 | 1 |
| Parent + Sibling diagnosis | 1 | 1 |

# SM2. Data availability and Inclusion/Exclusion comparison

| **Table S2.1** *Participants who provided data by Likelihood group and Timepoint.* | | | | | | | | | | | | | | | |
| --- | --- | --- | --- | --- | --- | --- | --- | --- | --- | --- | --- | --- | --- | --- | --- |
|  | **Attention data** | | | |  | **Movement data** | | | |  | **3-year CBCL data** | | | | |
|  |  | only | | both |  |  | only | | both |  |  | only | | both |  |
|  | Tot | 10m | 14m | 10 + 14m |  | Tot | 10m | 14m | 10 + 14m |  | Tot | 10m | 14m | 10 + 14 m |  |
|  | n | n | n | n (%) |  | n (%) | n | n | n (%) |  | n (%) | n | n | n (%) |  |
| TL | 26 | 8 | 6 | 12  (46 %) |  | 25 (96%) | 11 | 8 | 6  (24 %) |  | 20  (78%) | 6 | 6 | 8 (40%) |  |
| EL-autism | 70 | 14 | 15 | 41  (59 %) |  | 61 (87%) | 17 | 23 | 21  (34 %) |  | 53  (76 %) | 10 | 14 | 29 (55%) |  |
| EL-ADHD | 28 | 8 | 7 | 13  (46 %) |  | 26 (93%) | 10 | 9 | 7  (27 %) |  | 19  (68 %) | 4 | 4 | 11 (58%) |  |
| EL-autism  +ADHD | 18 | 2 | 5 | 11  (61 %) |  | 16 (89%) | 3 | 6 | 7  (44 %) |  | 13  (72 %) | 1 | 2 | 10 (77%) |  |
| NF1 | 29 | 8 | 8 | 13 (  45 %) |  | 25 (86%) | 9 | 8 | 8  (32 %) |  | 25  (86 %) | 7 | 7 | 11 (44%) |  |
| Total | 171 | 40 | 41 | 90  (53 %) |  | 153 (89%) | 50 | 54 | 49  (32 %) |  | 130  (76 %) | 28 | 33 | 69 (53%) |  |
| Note. Percentages for totals are given in relation to Attention total. Percentages for ‘10 + 14 month’ are in relation to totals for attention, movement and 3 year CBCL, respectively. | | | | | | | | | | | | | | |  |

| **Table S2.2** *Reasons for missing Puppet task data if attended infant visit at 10 and/or 14 months.* | | | | | | | | |
| --- | --- | --- | --- | --- | --- | --- | --- | --- |
| **Group** | **N** | **No visit** | **Parental interference** | **Technical issue** | **No puppet show** | **Baby too upset** | **Total**  **Exclude** | **Total Include**  **(%)** |
| **10 months** |  |  |  |  |  |  |  |  |
| TL | 27 | - | - | 5 | 1 | 1 | **7** | **20 (74%)** |
| EL-autism | 78 | 2 | 2 | 15 | 3 | 1 | **23** | **55 (71%)** |
| EL-ADHD | 31 | 4 | 1 | 4 | 1 | - | **10** | **21 (68%)** |
| EL-autism+ADHD | 20 | 1 | - | 6 | - | - | **7** | **13 (65%)** |
| NF1 | 31 | 8 | - | 2 | - | - | **10** | **21 (68%)** |
| *Total* | 187 | 10 | 3 | 32 | 5 | 2 | **57** | **130 (70%)** |
| **14 months** |  |  |  |  |  |  |  |  |
| TL | 27 | 4 | 3 | 1 | 1 | - | **9** | **18 (67%)** |
| EL-autism | 78 | 6 | 3 | 11 | 2 | - | **22** | **56 (72%)** |
| EL-ADHD | 31 | 6 | 1 | 3 | 1 | - | **11** | **20 (65%)** |
| EL-autism+ADHD | 20 | 1 | 1 | 1 | 1 | - | **4** | **16 (80%)** |
| NF1 | 31 | 5 | 1 | 2 | 1 | 1 | **10** | **21 (68%)** |
| *Total* | 187 | 22 | 9 | 18 | 6 | 1 | **56** | **131 (70%)** |

Chi-square test confirmed that inclusion versus exclusion rates for the Puppet task does not differ significantly by group at 10 months (X^2^ (4) = .59, *p* = .965) or 14 months (X^2^ (4) = 1.74, *p* = .784).

# SM3. Comparison of participants with and without data

| **Table S3.1** *Comparison of included and excluded participants who did attend visit.* | | | | | | | | |
| --- | --- | --- | --- | --- | --- | --- | --- | --- |
|  | **Included** | | | **Excluded** | | |  |  |
|  | **Mean** | **sd** | **n** | **Mean** | **sd** | **n** | **test** | ***p*** |
| **10 months**  Sex (m:f) | 71:59 |  |  | 21:21 |  |  | X^2^ = .27 | .602 |
| Age in days | 322.37 | 16.83 | 130 | 318.88 | 20.82 | 41 | *t* = 1.09 | .277 |
| Motor skills | 0.03 | 0.77 | 111 | 0.05 | 0.69 | 38 | *t* = 0.14 | .889 |
| Vineland ABC | 92.95 | 13.78 | 102 | 92.27 | 12.52 | 37 | *t* = -0.26 | .793 |
| **14 months**  Age in days | 450.31 | 20.05 | 130 | 448.03 | 19.93 | 32 | *t* = -0.58 | .565 |
| Motor skills | 0 | 0.71 | 113 | 0.23 | 0.70 | 29 | *t* = 1.52 | .130 |
| Vineland ABC | 93.81 | 11.32 | 108 | 99.03 | 10.73 | 29 | *t* = 2.23 | .028* |

**p<0.05*

| **Table S3.2** *Comparison of participants with and without accelerometer data.* | | | | | | | | |
| --- | --- | --- | --- | --- | --- | --- | --- | --- |
|  | **Included** | | | **Excluded** | | |  |  |
|  | **Mean** | **sd** | **n** | **Mean** | **sd** | **n** | **test** | ***p*** |
| **10 months** |  |  |  |  |  |  |  |  |
| Sex (m:f) | 52:47 |  |  | 19:12 |  |  | X^2^ = .73 | .392 |
| Age in days | 323.07 | 17.79 | 99 | 320.13 | 13.35 | 31 | *t* = -0.85 | .398 |
| Motor skills | 0.03 | 0.78 | 85 | 0.01 | 0.76 | 26 | *t* = -0.09 | .930 |
| Vineland ABC | 93.12 | 13.24 | 77 | 92.44 | 15.61 | 25 | *t* = -0.21 | .832 |
| **14 months** |  |  |  |  |  |  |  |  |
| Sex (m:f) | 59:44 |  |  | 11:17 |  |  | X^2^ = 2.87 | .091 |
| Age in days | 451.43 | 21.13 | 103 | 446.04 | 14.78 | 27 | *t* = -1.25 | .215 |
| Motor skills | -0.05 | 0.71 | 91 | 0.20 | 0.71 | 22 | *t* = 1.46 | .148 |
| Vineland ABC | 92.87 | 11.73 | 86 | 97.50 | 8.87 | 22 | *t* = 1.73 | .087 |

# SM4. Average duration of puppets across groups

| **Table S4.** *Average of the actual duration of each puppet and break period across all infants.* | | | | | | | | | | | | | |
| --- | --- | --- | --- | --- | --- | --- | --- | --- | --- | --- | --- | --- | --- |
|  | **S1** | **B1** | **D1** | **B2** | **D2** | **B3** | **D3** | **B4** | **D4** | **B5** | **D5** | **B6** | **S2** |
| Mean | 7.5 | 7.6 | 7.5 | 26.7 | 7.5 | 16.9 | 7.3 | 16.5 | 7.3 | 6.8 | 7.1 | 26.9 | 6.8 |
| SE(mean) | 0.1 | 0.1 | 0.1 | 0.2 | 0.1 | 0.1 | 0.1 | 0.1 | 0.1 | 0.1 | 0.1 | 0.2 | 0.1 |
| Min | 4.4 | 5.2 | 3.3 | 17.1 | 3.3 | 12.8 | 2.7 | 10.3 | 3.2 | 3.8 | 3.2 | 5.1 | 3.8 |
| Max | 11.9 | 14.9 | 11.9 | 39 | 10.8 | 27.6 | 10.6 | 24.5 | 11.4 | 12.9 | 11.5 | 39 | 10.8 |
| In grey: included in manuscript; S = Sprite, B = Break, D = Duck. | | | | | | | | | | | | | |

# SM5. Reliability of Attention behaviour coding

| **Table S5.** *Intraclass correlations for coded Attention behaviour (two-way mixed, single measures, absolute agreement).* | | | | | |
| --- | --- | --- | --- | --- | --- |
|  | **Puppet 1** | **Puppet 2** | **Puppet 3** | **Puppet 4** | **Puppet 5** |
| **10 months** |  |  |  |  |  |
| Puppet | .997 | .992 | .981 | .989 | .940 |
| Break | .935 | .916 | .994 | .992 | .947 |
| **14 months** |  |  |  |  |  |
| Puppet | .912 | .988 | .976 | .993 | .975 |
| Break | .908 | .944 | .969 | .950 | .970 |
| Note. Raters coded looking versus not looking at the puppet when the puppet was present (Puppet) and looking versus not looking at where the puppet was during the Break (Break) | | | | | |

# SM6. Summary of Attention and Movement variables (untransformed & transformed)

| **Table SM6.1** *Mean and standard deviation of proportion attending (0 to 1) and amount of movement (cm/s).* | | | | | | | | | | | | | | | | | | | | | | | | | | | | | | | | |
| --- | --- | --- | --- | --- | --- | --- | --- | --- | --- | --- | --- | --- | --- | --- | --- | --- | --- | --- | --- | --- | --- | --- | --- | --- | --- | --- | --- | --- | --- | --- | --- | --- |
|  | **Group** | | | | | | | | | | | | | | | | | | | | | | | | | | | | | | | |
|  | **EL-autism** | | | | | **EL-ADHD** | | | | **EL-autism+ADHD** | | | | | | | **NF1** | | | | | | | | **Typical Likelihood** | | | | | | | |
| **10 months** | M | (sd) | Min | Max | | M | (sd) | Min | Max | M | | (sd) | Min | | Max | | M | | (sd) | | Min | | Max | | M | | (sd) | | Min | | Max | |
| *Attention* |  |  |  |  | |  |  |  |  |  | |  |  | |  | |  | |  | |  | |  | |  | |  | |  | |  | |
| Focused Attention | 0.90 | (0.09) | 0.61 | 0.99 | | 0.90 | (0.08) | 0.72 | 0.99 | 0.86 | | (0.14) | 0.49 | | 1.00 | | 0.79 | | (0.15) | | 0.34 | | 0.93 | | 0.87 | | (0.13) | | 0.47 | | 1.00 | |
| Vigilance | 0.25 | (0.14) | 0.04 | 0.72 | | 0.21 | (0.13) | 0.04 | 0.46 | 0.22 | | (0.10) | 0.08 | | 0.44 | | 0.17 | | (0.11) | | 0.03 | | 0.50 | | 0.19 | | (0.10) | | 0.04 | | 0.40 | |
| *Movement* |  |  |  |  | |  |  |  |  |  | |  |  | |  | |  | |  | |  | |  | |  | |  | |  | |  | |
| Focused Attention | 4.07 | (7.30) | 0.00 | 34.75 | | 3.28 | (4.09) | 0.00 | 13.47 | 2.51 | | (3.60) | 0.00 | | 9.96 | | 5.34 | | (14.94) | | 0.00 | | 63.06 | | 2.74 | | (3.54) | | 0.00 | | 14.04 | |
| Vigilance | 5.87 | (8.08) | 0.00 | 27.86 | | 4.18 | (5.05) | 0.00 | 17.81 | 3.18 | | (3.66) | 0.00 | | 9.73 | | 7.48 | | (20.34) | | 0.00 | | 84.83 | | 4.38 | | (5.12) | | 0.00 | | 16.75 | |
| Looking Elsewhere | 9.11 | (10.41) | 0.34 | 43.75 | | 9.0 | (10.31) | 0.00 | 33.80 | 6.84 | | (5.76) | 0.13 | | 16.90 | | 6.51 | | (11.34) | | 0.00 | | 46.79 | | 8.40 | | (7.09) | | 0.23 | | 22.41 | |
| **14 months** | M | (sd) | Min | Max | | M | (sd) | Min | Max | M | | (sd) | Min | | Max | | M | | (sd) | | Min | | Max | | M | | (sd) | | Min | | Max | |
| *Attention* |  |  |  | |  |  |  |  |  |  |  | | |  | |  | |  | |  | |  | |  | |  | |  | |  | |  |
| Focused Attention | 0.89 | (0.11) | 0.47 | 1.00 | | 0.86 | (0.12) | 0.53 | 0.99 | 0.82 | | (0.14) | 0.39 | | 0.99 | | 0.81 | | (0.14) | | 0.53 | | 0.98 | | 0.85 | | (0.13) | | 0.52 | | 1.00 | |
| Vigilance | 0.20 | (0.11) | 0.03 | 0.50 | | 0.20 | (0.14) | 0.01 | 0.60 | 0.20 | | (0.12) | 0.08 | | 0.57 | | 0.16 | | (0.09) | | 0.04 | | 0.38 | | 0.18 | | (0.07) | | 0.07 | | 0.31 | |
| *Movement* |  |  |  |  | |  |  |  |  |  | |  |  | |  | |  | |  | |  | |  | |  | |  | |  | |  | |
| Focused Attention | 2.83 | (5.62) | 0.00 | 25.59 | | 4.94 | (8.18) | 0.00 | 24.85 | 3.10 | | (5.55) | 0.00 | | 19.62 | | 3.87 | | (9.50) | | 0.00 | | 38.82 | | 3.95 | | (6.05) | | 0.00 | | 18.49 | |
| Vigilance | 5.27 | (10.95) | 0.00 | 58.33 | | 4.94 | (8.86) | 0.00 | 33.99 | 4.22 | | (5.83) | 0.00 | | 17.83 | | 3.56 | | (5.14) | | 0.00 | | 17.20 | | 4.50 | | (8.34) | | 0.00 | | 32.09 | |
| Looking Elsewhere | 6.61 | (9.62) | 0.00 | 38.61 | | 9.58 | (15.62) | 0.04 | 61.66 | 6.48 | | (6.81) | 0.60 | | 22.76 | | 7.04 | | (5.57) | | 0.13 | | 19.79 | | 9.10 | | (10.42) | | 0.01 | | 32.69 | |

Attention data was transformed using a logit transformation; Movement data was transformed using a natural log transformation. Before transformation, 0 and 1 values in Attention and Movement data were adjusted by adding or subtracting .01. Transformation improved the normal distribution of the data in general, however the cases previously having 0 or 1 values were still observed as outliers.

| **Table SM6.2** *Mean (standard deviation) of proportion Attending and amount of Movement (cm/s), adjusted and transformed.* | | | | | | | | | | |
| --- | --- | --- | --- | --- | --- | --- | --- | --- | --- | --- |
| **Likelihood Group** | | | | | | | | | | |
|  | **EL-autism** | | **EL-ADHD** | | **ELautism+ADHD** | | **NF1** | | **TL** | |
|  | **Mean** | **(sd)** | **Mean** | **(sd)** | **Mean** | **(sd)** | **Mean** | **(sd)** | **Mean** | **(sd)** |
| **10 Months** |  |  |  |  |  |  |  |  |  |  |
| Attention |  |  |  |  |  |  |  |  |  |  |
| Focused Attention | 2.62 | (1.16) | 2.69 | (1.29) | 2.52 | (1.71) | 1.49 | (0.84) | 2.48 | (1.42) |
| Vigilance | -1.24 | (0.77) | -1.52 | (0.86) | -1.37 | (0.58) | -1.81 | (0.84) | -1.57 | (0.71) |
| Movement |  |  |  |  |  |  |  |  |  |  |
| Focused Attention | -0.37 | (2.35) | -0.51 | (2.67) | -0.93 | (2.75) | -0.11 | (2.28) | -0.19 | (2.12) |
| Vigilance | 0.50 | (1.95) | -0.04 | (2.55) | -0.02 | (2.15) | 0.18 | (2.10) | 0.51 | (1.92) |
| Looking Elsewhere | 1.60 | (1.19) | 1.13 | (2.17) | 1.28 | (1.54) | 0.83 | (1.80) | 1.64 | (1.21) |
| **14 Months** |  |  |  |  |  |  |  |  |  |  |
| Attention |  |  |  |  |  |  |  |  |  |  |
| Focused Attention | 2.78 | (1.38) | 2.24 | (1.19) | 1.86 | (1.24) | 1.67 | (0.97) | 2.31 | (1.52) |
| Vigilance | -1.54 | (0.74) | -1.60 | (1.00) | -1.48 | (0.66) | -1.84 | (0.69) | -1.59 | (0.50) |
| Movement |  |  |  |  |  |  |  |  |  |  |
| Focused Attention | -1.04 | (2.42) | -0.58 | (2.64) | -0.73 | (2.49) | -0.38 | (2.18) | -0.41 | (2.48) |
| Vigilance | -0.16 | (2.51) | -0.19 | (2.60) | -0.17 | (2.57) | -0.49 | (2.61) | -0.35 | (2.62) |
| Looking Elsewhere | 0.84 | (1.88) | 1.15 | (1.79) | 1.22 | (1.28) | 1.48 | (1.26) | 1.00 | (2.29) |

# SM7. Mullen Scales of Early Learning

The Mullen Scales of Early Learning (1) was administered at all time points by trained researchers in the STAARS team. To allow for the greatest level of replicability and consistency across examiners, we have extremely strict guidelines about how the Mullen should be administered and marked (2). To this end, our guidelines for Mullen scoring include only behaviours that are captured on camera (so can be confirmed by a second/third researcher if necessary) within the Mullen session. For example, if an infant demonstrates babbling throughout the rest of the testing day (i.e., during another task or a lunch break), but not during the specific Mullen administration session, we would not score this infant as being able to produce babbling sounds on the Expressive Language scale. To further ensure the fidelity of the scoring, a second fully trained researcher watches the administration in real time (via a video feed) and consensus discussions take place after the testing session. These strict administration and scoring guidelines (although those recommended in the Mullen manual) may not be those applied more broadly in the field and thus may account for relatively poorer performance in this cohort at infant timepoints relative to US norms.

# SM8. Correlations of Mullen and Vineland Motor scales

**Table S8.1.** *Correlations of Motor variables and with Combined Motor Skill Scale.*

| **10 months** | **N** | **MSEL GM** | **MSEL FM** | **VABS Motor** |
| --- | --- | --- | --- | --- |
| MSEL GM | 130 |  |  |  |
| MSEL FM | 130 | 0.38 (<.001) |  |  |
| VABS Motor | 111 | 0.49 (<.001) | 0.40 (<.001) |  |
| Combined Motor Skill Score | 111 | 0.77 (<.001) | 0.76 (<.001) | 0.81 (<.001) |
| **14 months** |  |  |  |  |
| MSEL GM | 130 |  |  |  |
| MSEL FM | 130 | 0.21 (.015) |  |  |
| VABS Motor | 113 | 0.43 (<.001) | 0.19 (.041) |  |
| Combined Motor Skill Score | 113 | 0.75 (<.001) | 0.64 (<.001) | 0.76 (<.001) |
| MSEL: Mullen Scales of Early Learning, GM: Gross Motor, FM: Fine Motor, VABS: Vineland Adaptive Behavior Scale | | | | |

# SM9. Model effects and interactions for Transformed Attention variable using Linear Mixed Model

To confirm the robustness of the results of the GLMM, Linear mixed models were run on the transformed Attention data, using robust standard errors and an independent residual structure. Predictor variables were Condition, Group, Timepoint and their interaction. The overall model was significant (Wald X^2^(19) = 2726.39, *p*<.001). Results of the model and the ‘*margins, pwcompare*’ command can be found in Table S11.1 and Table S11.2 and showed a significant effect of Condition (*p* < .001), Group (*p* < .001) and a Condition*Group interaction (*p*=.003). The main effect of Condition explained an additional 77.0% of the variance, whilst adding Group explained 2.1% (Group main effect only: 1.5%).

Comparing Bonferroni corrected margins for the main effect of Group (see also Table S11.2), the NF1 (Estimated Marginal Mean (EMM) = -0.14, SE = 0.12) group looked less compared to TL (EMM = 0.42, SE = 0.14; *p* = .020), the EL-autism (EMM = 0.66, SE = .09; *p* < .001) and EL-ADHD groups (EMM = 0.45, SE = 0.13; *p* = .010). As in the GLMM model, and against our hypothesis, the other EL groups did not differ from the TL group or each other.

To examine the interaction effect, we compared margins for Focused Attention and Vigilance separately, which showed that similar results for the Focused Attention condition (NF1 group attended less compared to the TL, EL-autism and EL-ADHD groups), but during Vigilance the NF1 group (EMM = -1.84, SE = 0.12) showed less Attention compared to the EL-autism group (EL-autism: EMM = -1.39, SE = 0.08, *p* = .014) but no difference compared to the TL (EMM = -1.57, SE = 0.11; *p* = .956) or EL-ADHD (EMM = -1.56, SE = 15; *p* = 1) groups.

Removing 2 outliers changed the results slightly. In addition to the previous results, the EL-autism+ADHD (EMM = 0.28, SE = 0.15) showed less Focused Attention compared to the EL-autism group (EMM = 0.65, SE = 0.09; *p* = .045). The NF1 group (EMM = -.15, SE = 0.12) no longer differed significantly from the TL group (EMM = .37, SE = 0.14, *p* = .065). For Vigilance, the results remained the same.

Thus, overall results were comparable to the GLMM model, with the most consistent finding that of the NF1 group compared to the EL-autism and EL-ADHD groups during Focused Attention, and NF1 vs. EL-autism group during Vigilance.

**Table S9.1** *Model statistics of linear mixed model for Looking.*

| **Effect** | **Estimate** | **SE** | **df** | **𝜒2** | **p** | **95% CI** |
| --- | --- | --- | --- | --- | --- | --- |
| Group | - | - | 4 | 29.45 | < .001 | - |
| Condition | - | - | 1 | 1866.12 | < .001 | - |
| Timepoint | - | - | 1 | 1.35 | .246 | - |
| Group x Condition | - | - | 4 | 16.37 | .003 |  |
| Group x Timepoint | - | - | 4 | 1.57 | .813 | - |
| Condition x Timepoint | - | - | 1 | 0.07 | .791 | - |
| Group x Condition x Timepoint | - | - | 4 | 5.47 | .243 | - |
|  | **Estimate** | **SE** | **-** | **z** | **p** | **95% CI** |
| **Group** |  |  |  |  |  |  |
| TL | 0.42 | 0.14 | - | 3.04 | .002 | [0.15, 0.69] |
| EL-autism | 0.66 | 0.09 | - | 7.56 | < .001 | [0.49, 0.83] |
| EL-ADHD | 0.45 | 0.13 | - | 3.38 | .001 | [0.19. 0.71] |
| EL-autism+ADHD | 0.34 | 0.15 | - | 2.28 | .023 | [0.05, 0.63] |
| NF1 | -0.14 | 0.12 | - | -1.15 | .252 | [-0.37. 0.10] |
| **Condition** |  |  |  |  |  |  |
| Focused Attention | 2.37 | 0.08 | - | 29.27 | < .001 | [2.21. 2.53] |
| Vigilance | -1.52 | 0.05 | - | -31.19 | < .001 | [-1.61, -1.42] |
| **Timepoint** |  |  |  |  |  |  |
| 10m | 0.48 | 0.07 | - | 6.83 | < .001 | [0.34, 0.61] |
| 14m | 0.38 | 0.07 | - | 5.30 | < .001 | [0.24, 0.51] |

**Table 9.2** *Bonferroni comparison of significant main Group and main Condition effects.*

|  | **Contrast** | **SE** | z | **(adj) p** | **95% CI** |
| --- | --- | --- | --- | --- | --- |
| **Group Comparison** |  |  |  |  |  |
| EL-autism vs TL | 0.23 | 0.16 | 1.47 | 1 | [-0.22, 0.69] |
| EL-ADHD vs TL | 0.03 | 0.19 | 0.18 | 1 | [-0.50, 0.57] |
| EL-A+A vs TL | -0.08 | 0.20 | -0.39 | 1 | [-0.64, 0.49] |
| NF1 vs TL | -0.55 | 0.18 | -3.05 | .023 | [-1.06, -0.04] |
| EL-ADHD vs EL-autism | -0.21 | 0.16 | -1.29 | 1 | [-0.65, 0.24] |
| EL-autism+ADHD vs EL-autism | -0.32 | 0.17 | -1.86 | .634 | [-0.80, 0.16] |
| NF1 vs EL-autism | -0.79 | 0.15 | -5.39 | < .001 | [-1.20, -0.38] |
| EL-autism+ADHD vs EL-ADHD | -0.11 | 0.20 | -0.57 | 1 | [-0.67, 0.45] |
| NF1 vs EL-ADHD | -0.59 | 0.18 | -3.29 | .010 | [-1.09, -0.09] |
| NF1 vs EL-autism+ADHD | -0.47 | 0.19 | -2.49 | .127 | [-1.01, 0.06] |
| **Condition Comparison** |  |  |  |  |  |
| Vigilance vs FA | -3.89 | 0.08 | -48.10 | < .001 | [-4.05, -3.73] |

# SM10. Effect of position

| **Table S10.1** *Number of infants sitting on Highchair vs Lap per Timepoint.* | | | | |
| --- | --- | --- | --- | --- |
|  | **10 months** | | **14 months** | |
| **Group** | **Highchair** | **Lap** | **Highchair** | **Lap** |
| TL | 13 | 4 | 8 | 6 |
| EL-autism | 30 | 8 | 34 | 10 |
| EL-ADHD | 12 | 5 | 11 | 5 |
| EL-autism+ADHD | 8 | 2 | 10 | 3 |
| NF1 | 13 | 4 | 12 | 4 |
| **Total** | **76** | **23** | **75** | **28** |

There were no differences between Groups in the number of infants sitting in a highchair versus on the lap (10 months: $\chi^{2}$ = 0.53, *p* = .971; 14 months: $\chi^{2}$ = 2.46, *p* = .651). To examine the influence of Position on Attention and Movement, we conducted separated ANOVAs for the 10- and 14- month assessments using the transformed Attention and Movement data. The first ANOVA included only Position, the second included Position-by-Condition and the third Position-by-Group.

We found no Position, Position-by-Condition or Position-by-Group interaction effect on Attention behaviour at either Timepoint (see Table S8.2). For Movement, there was a main effect of Position, with infants moving more when on the parent’s lap compared to the highchair. There was no interaction with Condition at either Timepoint, but there was a Position-by-Group interaction at 14 months.

| **Table S10.2** *Anova results to look at the effect of Position on Attention and Movement data.* | | | | | | |
| --- | --- | --- | --- | --- | --- | --- |
|  | **10 months** | | | **14 months** | | |
| **Attention** | **F** | **df** | ***p*** | **F** | **df** | ***p*** |
| Position | 0.08 | 1 | .782 | 0.45 | 1 | .504 |
| Position x Condition | 0.17 | 1 | .684 | 0.83 | 1 | .362 |
| Position x Group | 0.16 | 4 | .957 | 0.09 | 4 | .984 |
| **Movement** |  |  |  |  |  |  |
| Position | 26.45 | 1 | < .001 | 14.50 | 1 | < .001 |
| Position x Condition | 1.84 | 2 | .162 | 0.90 | 2 | .408 |
| Position x Group | 0.32 | 4 | .865 | 4.91 | 4 | < .001 |

To examine the Position-by-Group effect, we followed up with a margins command (Figure S8). This showed that the differences in Movement between the lap and highchair were more pronounced in the EL-autism (Contrast = 2.88, SE = .85, t = 3.39, *p* = .008) and the EL-ADHD groups (Contrast = 3.61, SE = 1.00, t = 3.61, *p* = .004), compared to the TL group (after Bonferroni correction). None of the other contrasts was significant.

| 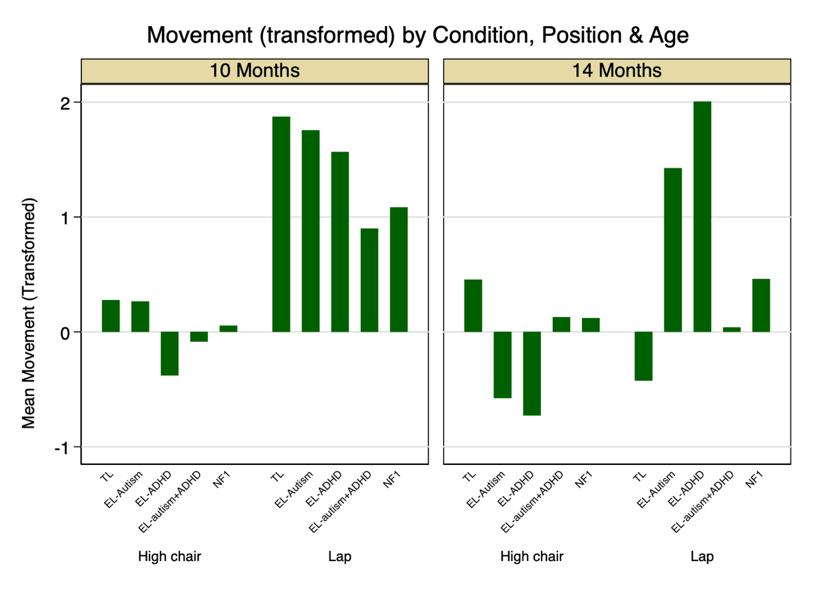 |
| --- |
| **Figure S10.** *Bar graphs of Movement (transformed) by Position and Group and Timepoint.* |

# SM11. Additional information for Attention GLMM Model

**Table SM11.1** *Bonferroni comparison of significant model effects.*

| **Group Comparison** | **Estimate** | **SE** | **df** | **t** | **(adj) p** | **95% CI** |
| --- | --- | --- | --- | --- | --- | --- |
| TL vs EL-autism | -0.27 | 0.13 | 170 | -2.16 | .322 | [-0.63, 0.09] |
| TL vs EL-ADHD | -0.15 | 0.15 | 170 | -0.98 | 1 | [-0.57, 0.28] |
| TL vs EL-autism+ADHD | 0.04 | 0.16 | 170 | 0.26 | 1 | [-0.41, 0.50] |
| TL vs NF1 | 0.34 | 0.14 | 170 | 2.40 | .173 | [-0.06, 0.75] |
| EL-autism vs EL-ADHD | 0.13 | 0.12 | 170 | 1.04 | 1 | [-0.22, 0.48] |
| EL-autism vs EL-autism+ADHD | 0.32 | 0.14 | 170 | 2.31 | .221 | [-0.07, 0.70] |
| EL-autism vs NF1 | 0.62 | 0.11 | 170 | 5.43 | < .001*** | [0.29, 0.94] |
| EL-ADHD vs EL-autism+ADHD | 0.19 | 0.16 | 170 | 1.19 | 1 | [-0.26, 0.64] |
| EL-ADHD vs NF1 | 0.49 | 0.14 | 170 | 3.53 | .005** | [0.09, 0.88] |
| EL-autism+ADHD vs NF1 | 0.30 | 0.15 | 170 | 1.98 | .498 | [-0.13, 0.73] |

| **Table SM11.2** *Odds ratios GLM beta regression model.* | | | |
| --- | --- | --- | --- |
|  | **Estimate OR** | **df** | **95% CI** |
| **Groups** |  |  |  |
| TL vs NF1 | 1.40 | 170 | [1.06, 1.87] |
| EL-ADHD vs NF1 | 1.63 | 170 | [1.24, 2.14] |
| EL-autism vs NF1 | 1.85 | 170 | [1.48, 2.32] |
| EL-autism+ADHD vs NF1 | 1.35 | 170 | [1.00, 1.82] |
| **Conditions** |  |  |  |
| Focused Attention vs Vigilance | 24.39 | 170 | [20.46, 29.09] |

# SM12. Additional Data for Movement Model

**Table S12.** *Bonferroni results of significant main effects in Movement model.*

|  | **Estimate** | **SE** | **z** | ***p*** | **95% CI** |
| --- | --- | --- | --- | --- | --- |
| **Movement Comparison** |  |  |  |  |  |
| Vigilance vs FA | 0.59 | 0.13 | 4.42 | < .001*** | [0.27, 0.90] |
| Elsewhere vs FA | 1.77 | 0.12 | 14.54 | < .001*** | [1.48, 2.06] |
| Elsewhere vs Vigilance | 1.18 | 0.11 | 11.06 | < .001*** | [0.93, 1.44] |
| **Position Comparison** |  |  |  |  |  |
| Lap vs Highchair | 1.49 | 0.33 | 4.51 | < .001*** | [0.84, 2.14] |

*Mixed models By Position*

*Highchair.* There was a significant of Condition ($\chi^{2}$(2) = 159.16, *p* < .001, and posthoc comparison showed a similar Condition effect (Focused Attention < Vigilance < Elsewhere) with all contrasts different (all *p*’s < .001).

*Lap.* There was a significant of Condition ($\chi^{2}$(2) = 134.11, *p* < .001, however post hoc comparison showed there was no significant differences between Focused Attention and Vigilance (Contrast = -0.12, SE = 0.23, *z* = -0.53, *p* = 1) whereas movement was higher during looking Elsewhere compared to both Focused Attention and Vigilance. There was also a significant three-way interaction between Condition, Group and Timepoint ($\chi^{2}$(2) = 134.11, *p* < .001) and inspecting the margins plot, showed that especially at 14 months, there was more variability in movement across Groups and Conditions (see Figure S12), however it should be noted that there were less children seated on the lap versus highchair (see also S8.1)

| **a.**  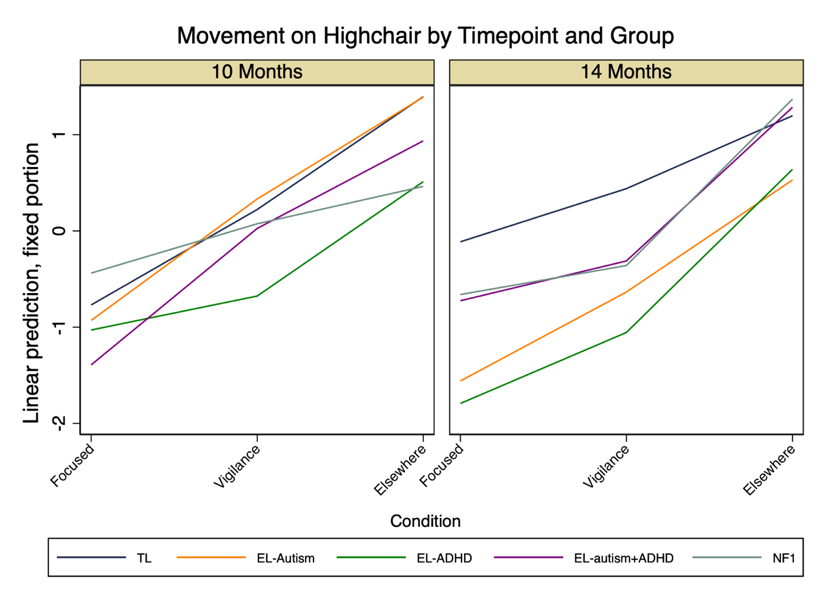 |
| --- |
| **b.**  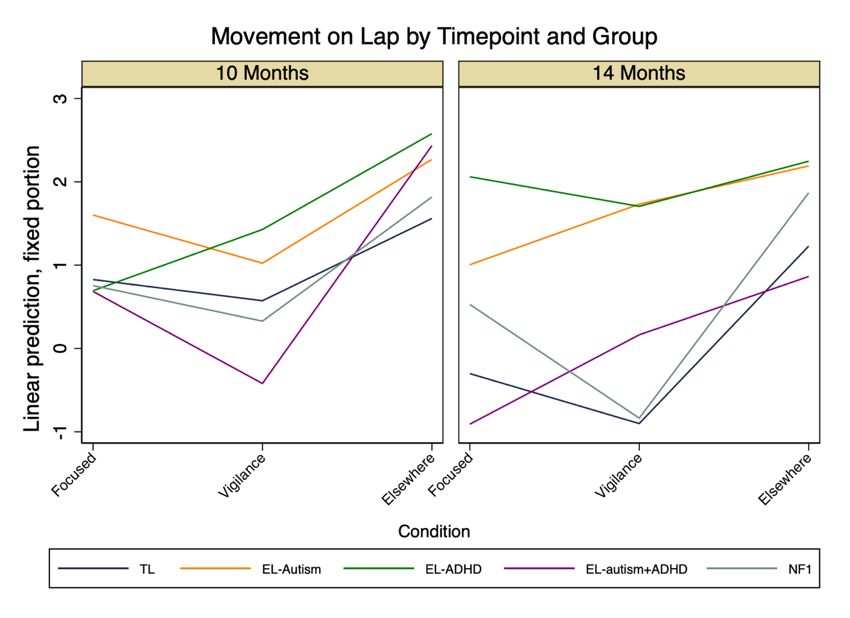 |

| **Figure S12.** *Movement per position (a. highchair, b. lap) and by Timepoint, Group and Condition.* |
| --- |

*Non-parametric Kruskal-Wallis results*

Considering the deviations in fit in the main model, we ran a non-parametric Kruskal-Wallis test to confirm our results. This test showed the same significant effect of Condition ($\chi^{2}$(2) = 66.23, *p* <.001), but not Group ($\chi^{2}$(4) = 1.03, *p* = .906). All Conditions significantly differed from each other after Bonferroni correction: Focused Attention (Med = 0.69, IQR= 3.36), vs Vigilance (Med = 1.69, IQR= 4.97; *z* = -2.54, *p* = .017); Focused Attention vs Looking Elsewhere (Med = 4.34, IQ = 8.3; *z* = -7.97, *p* < .001); Vigilance vs Looking Elsewhere (*z* = -5.43, *p* < .001).

# SM13. Associations between Attention and Movements and ADHD traits at 3 years.

**
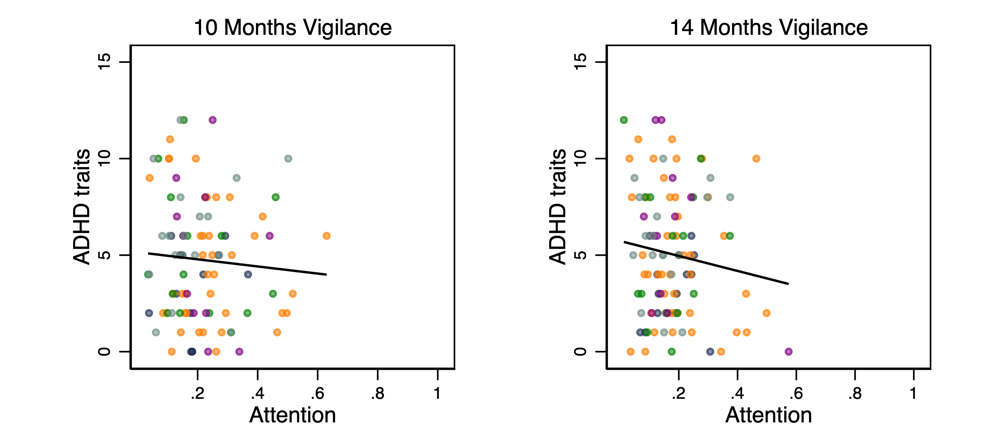
**

**
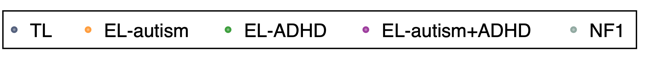
**

**Figure S13.1** *Scatterplot of 10 and 14 month Vigilance with 3 year CBCL-ADHD traits.*

**
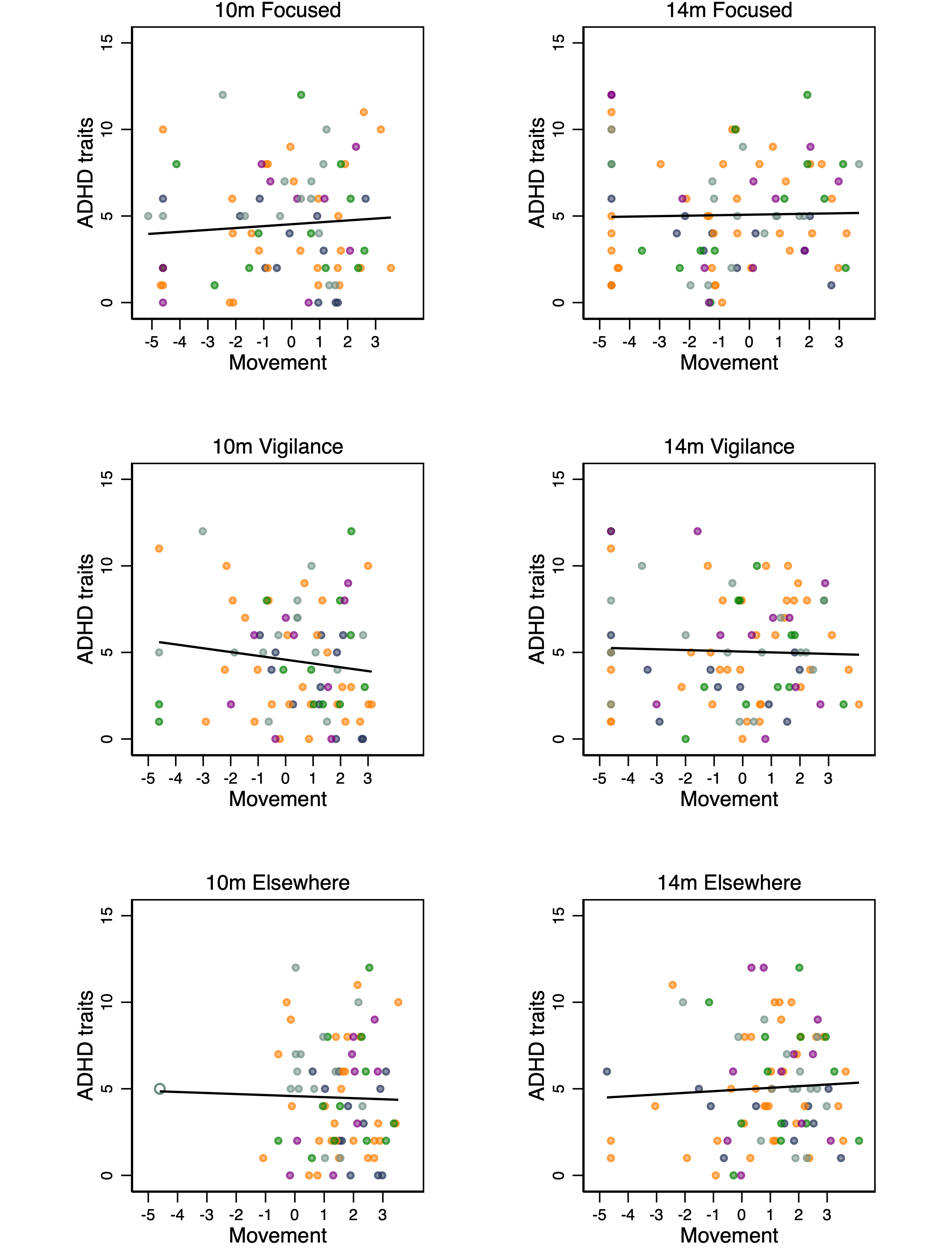
**

**
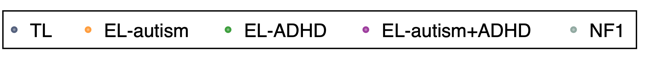
**

**Figure S13.2.** *Scatterplot of 10 and 14 month Movement (transformed) with 3 year CBCL-ADHD traits. Visual outliers plotted as open circles.*

*Attention-by-Movement Interaction.*

| **Table S13.1** *Model fit of Negative Binomial Interaction models Condition and Timepoint.* | | | | |
| --- | --- | --- | --- | --- |
|  | **n** | **Wald** $\boldsymbol{\chi}^{\boldsymbol{2}}$ | **Df** | ***p*** |
| **10 months** |  |  |  |  |
| Focused Attention | 75 | 6.20 | 3 | .102 |
| Vigilance | 75 | 5.19 | 3 | .158 |
| **14 months** |  |  |  |  |
| Focused Attention | 85 | 4.89 | 3 | .180 |
| Vigilance | 85 | 20.77 | 3 | < .001 |

| **Table S13.2** *Model Coefficients for Vigilance Binomial Interaction model at 14 months.* | | | | | |
| --- | --- | --- | --- | --- | --- |
|  | **Coefficient** | **SE robust** | **z** | ***p*** | **95% CI** |
| Attention | -0.85 | .67 | -1.26 | .206 | [-2.17, 0.47] |
| Movement | 0.003 | .02 | 0.15 | .882 | [-0.04, 0.05] |
| Attention x Movement | 0.75 | .21 | 3.57 | < .001*** | [0.34, 1.17] |
| *Added second model* |  |  |  |  |  |
| Position (Lap) | -0.12 | .14 | -0.87 | .383 | [-0.40, 0.16] |
| Motor skills | -0.15 | .10 | -1.46 | .144 | [-0.36, 0.05] |

# References

1. Mullen E. Mullen scales of early learning. American Guidance Service; 1995.

2. Begum Ali J, Charman T, Johnson MH, Jones EJH, and the BASIS/STAARS Team. Early Motor Differences in Infants at Elevated Likelihood of Autism Spectrum Disorder and/or Attention Deficit Hyperactivity Disorder. J Autism Dev Disord. 2020 Dec;50(12):4367–84.
